# Helminth-infected Mozambican children with malaria have increased anaemia, cytokines and helminth-specific antibodies

**DOI:** 10.1371/journal.pntd.0014485

**Published:** 2026-07-13

**Authors:** Inocência Cuamba, Rebeca Santano, Berta Grau-Pujol, Marta Vidal, Anélsio Cossa, Chenjerai Jairoce, Rojelio Mejía, José Muñoz, Ruth Aguilar, Bin Zhan, Augusto Nhabomba, Gemma Moncunill, Carlota Dobaño

**Affiliations:** 1 Centro de Investigação em Saúde de Manhiça (CISM), Manhica, Mozambique; 2 ISGlobal, Barcelona, Spain; 3 Facultat de Medicina i Ciències de la Salut, Universitat de Barcelona (UB), Barcelona, Spain; 4 CIBER Enfermedades Infecciosas (CIBERINFEC), Barcelona, Spain; 5 Baylor College of Medicine, Houston, Texas, United States of America; University of Calgary, CANADA

## Abstract

Owing to their overlapping geographical distribution and the consequent occurrence of coinfections, several studies have evaluated the impact of helminth infections on malaria immune responses and clinical outcomes. However, little has been reported on how malaria coinfection affects anthelmintic responses in children harbouring worm infections. We therefore aimed to assess the impact of malaria coinfection on helminth-related clinical and immune outcomes in children. Here, we measured in plasma the levels of 30 cytokines, total IgE, and helminth-specific IgM, IgA, IgG, IgG1-4, and IgE antibodies to a panel of 11 helminth antigens by Luminex. Samples were obtained from 441 children aged 2–10 years with diverse symptomatology, recruited from two hospitals in the Manhiça District (Mozambique). Parasite infections were diagnosed by rapid antigen diagnostic test, microscopy, and/or quantitative PCR. Among the recruited children, 96 were diagnosed with helminth infection, of whom 16 (17%) were coinfected with *Plasmodium falciparum,* and 80 (83%) had helminth infection alone. Coinfection was associated with an increased prevalence of anaemia (33% [5/15], p = 0.006) compared to helminth infection alone. Coinfected children also exhibited higher concentrations of pro-inflammatory cytokines (IL-2R and IL-6), cell-recruiting chemokines (MCP-1 and MIG), anti-inflammatory proteins (IL-1RA and IL-10), and the growth factor HGF (p < 0.05). Additionally, helminth-specific antibody responses were significantly enhanced (p < 0.05) in coinfected participants, with total IgG and IgM responses showing the strongest associations with coinfection. Notably, antibodies to several antigens, including Sh-SERPIN, Sm-SERPIN, As-37, Na-SAA-2, Na-GST-1, and Tm-16, were consistently increased across multiple isotypes (IgG, IgG cytophilic subclasses, IgM, and IgE) among coinfected children. In summary, malaria coinfection was associated with an increased risk of anaemia and enhanced inflammation and specific antibody responses in helminth-infected children, which could potentially influence worm expulsion or survival, as well as helminth vaccine efficacy.

## Author summary

In many low-income regions of the world, children are exposed to multiple infections, including neglected tropical diseases (NTDs), at a time when their immune system is still maturing. Due to their overlapping distributions, malaria and worms are among the most common co-occurring infections. The co-occurrence of these parasitic diseases could influence how each illness progresses and how the body’s immune system responds to either of the infections. While several studies have evaluated the impact of worm infections on malaria immune response, less is known about how malaria may alter the body’s response to worms. In our study, we recruited children from southern Mozambique who were infected with worms, some of whom also had malaria. We found that children with both infections were more likely to have anaemia than those infected only with worms. Coinfected children also showed higher levels of immune mediators in their blood, and stronger antibody responses to worm antigens, compared to children with worm infections alone. These results suggest that malaria can alter the immune response to worms and worsen anaemia. This interaction may affect how worms survive or are cleared by the immune system, and could also potentially influence how individuals respond to future vaccines against NTDs.

## Introduction

In 2023, 1.495 billion people worldwide required interventions against neglected tropical diseases (NTDs), particularly in tropical and subtropical regions [1], making them a significant global health issue. The World Health Organisation (WHO) currently recognises 21 major NTDs, which include helminthic infections such as schistosomiasis and soil-transmitted helminthiases (STH; *Ascaris lumbricoides*, *Ancylostoma duodenale*, *Necator americanus*, *Trichuris trichiura*, and *Strongyloides stercoralis*) [2]. Although these helminth infections rarely result in death, they contribute to substantial morbidity, which highly depends on the parasite burden. Clinical manifestations range from asymptomatic cases in low-intensity infections to a variety of signs and symptoms in high-intensity infections, including diarrhoea, abdominal pain, malnutrition, anaemia, general weakness, stunted growth, and delayed physical development [3–5].

Helminth-infected individuals in malaria and helminth coendemic regions can often present coinfection with malaria parasites [6,7]. Similar to helminth infections, malaria predominantly occurs in tropical areas, with an estimated 282 million cases worldwide in 2024, when Mozambique ranked among the top five countries with the highest burden [8]. The most prevalent malaria species is *Plasmodium falciparum*, and infection is clinically characterised by the presentation of fever, headache, and chills, and it significantly contributes to anaemia [8–10].

Several studies have highlighted the possible interactions between *P. falciparum* and helminth parasites during concurrent infections [6,11,12], particularly considering the opposing immune mechanisms induced by these pathogens. Acute malaria induces T helper (Th)1 cell responses [13,14], which can suppress Th2 responses that are critical for helminth control and clearance [15]. Additionally, the regulatory cytokine interleukin (IL)-10 may increase after malaria infection as a response to the malaria-induced pro-inflammatory environment [16], thereby dampening both Th1 and Th2 responses, creating an environment that favours the survival of both *P. falciparum* and helminths. Notably, helminths also induce regulatory cytokines, such as IL-10 and TGFβ, to support their survival in the host [16]. We hypothesize that these immunological interactions during malaria-helminth coinfections may alter immunological, parasitological and clinical outcomes.

One of the most commonly evaluated clinical outcomes of malaria-helminth coinfections is anaemia, with studies reporting varying findings. Coinfections have been associated with both increased [11,17,18] and reduced anaemia [19,20], suggesting a potential impact of coinfection, although without a clear consensus on the direction of the effect. Furthermore, in mouse models, malaria-helminth coinfections have been associated with exacerbated helminth morbidity by altering immune responses involved in worm clearance. For example, simultaneous acute infection with *Nippostrongylus brasiliensis* and *Plasmodium chabaudi* altered both local and lymph node type 2 immune activation [21]. *P. chabaudi* infection also significantly suppressed Th2 cytokine responses upon stimulation with *Schistosoma mansoni* soluble egg antigen (SEA), as well as anti-SEA IgM and IgG responses, for up to 1 month after the malaria infection [22]. This suppressive effect could potentially interfere with helminth clearance. Furthermore, there has been increasing interest in developing helminth vaccines as an additional control measure in the push toward helminth elimination. This has thus far proven challenging due to the complexity of these pathogens and host-parasite interactions [23]. It is thought that an ideal STH vaccine should elicit a robust Th2 immune response [24]. Given that these vaccines are being developed for use in countries endemic for several Th1-inducing infections, including malaria, it is essential to determine whether coinfection could also suppress vaccine responses to worm antigens.

Controversially, in humans, a potential protective effect resulting from malaria coinfection has been suggested. In two studies, children coinfected with malaria and schistosomiasis had enhanced anti-schistosome IgE and IgG3 responses compared to children with schistosomiasis but negative for malaria [25,26].

The impact of malaria on helminth immune responses and clinical outcomes is likely determined by the extent to which *Plasmodium*-driven inflammation disrupts the Th2/regulatory milieu required for effective anti-helminth immunity. The direction and magnitude of this interaction are likely shaped by factors such as helminth species, parasite burden, infection chronicity, host age, and transmission intensity [27]. Inconsistencies across studies may further reflect variations in study design, diagnostic methods, and failure to control for confounding variables, such as socio-economic factors [11,27].

Most existing research has focused on the impact of helminth infections on other diseases, including malaria, while the effects of coinfection on helminthiases per se have been largely neglected, and primarily conducted in murine models. Human studies have mainly focused on the effect of malaria on schistosomiasis [25,26], leaving a gap regarding the impact on STH infections. Both STH infections and schistosomiasis are characterised by predominantly Th2-biased and regulatory immune responses during chronic infection; however, schistosomiasis typically exhibits a more pronounced biphasic response, with an early Th1 phase followed by a strong Th2 shift driven by egg deposition [28,29]. Therefore, species-specific studies are needed, as the consequences may differ. Additionally, there is no consensus on the impact of malaria coinfection on the outcomes of helminth infections and helminth-specific immune responses. Such information is essential in supporting the implementation of integrated disease control programs, as recommended by the 2021–2030 roadmap for NTDs [30], as well as for the rational design and delivery of future helminth vaccines and programs.

Given the potential impact of malaria coinfection on helminth immune responses, we hypothesised that the Th1-dominant immune environment induced by *Plasmodium* infection would suppress Th2-associated anti-helminth responses in children, including helminth-specific IgE and IgG4 antibody levels and type 2 cytokine concentrations, while elevating pro-inflammatory cytokines. We also hypothesised that these immunological alterations would be accompanied by worsened clinical outcomes compared to children with helminth infection alone. This work builds upon a previous community-based study where we analysed the effect of malaria coinfection/coexposure on helminth-specific antibody responses [31]. However, those analyses were limited to only IgG and total IgE responses. To test these hypotheses, we measured plasma concentrations of 30 cytokines, chemokines and growth factors, as well as the levels of total IgE and helminth-specific IgM, IgA, IgG, IgG1-4, and IgE antibodies, comparing these biomarkers between infection groups. In our antigen panel, we included schistosomiasis and STH targets that are considered potential vaccine candidates.

## Methods

### Ethics statement

Written informed consent was obtained from parents or guardians. The protocol was approved by the Mozambican National Bioethics Committee (134/CNBS/16) and the Clinical Research Ethics Committee in Spain (HCB/2016/0447).

### Study design

The present research was conducted as part of the EcoHeMa project, a hospital-based cross-sectional study carried out in the Manhiça District, in southern Mozambique. Between August 2015 and December 2016, a total of 441 children aged 2–10 years presenting with several mild symptoms, such as fever, cough, diarrhoea and vomiting, were recruited at the Manhiça District Hospital and the Ilha Josina Machel Health Centre. This study was designed as an exploratory investigation within the EcoHeMa project, therefore, the sample size was determined by the number of suitable samples available from enrolled participants. Additionally, the age group was defined to address the objectives of the main epidemiological project, which hypothesised that children aged 2–10 years are potentially the most exposed due to their behavioural characteristics. Children who had taken antimalarial or anthelmintic medication within the preceding month were not eligible for inclusion.

At enrollment, 6 mL of venous blood was collected for malaria rapid diagnostic testing, hematocrit assessment, and HIV serology. The remaining blood was preserved as dried blood spots (DBS), and centrifuged to separate plasma that was stored at -80°C at the laboratory of the Centro de Investigação em Saúde de Manhiça (CISM). Stool samples were obtained the following day during household visits by field workers and transported to CISM under cooled conditions (2–8°C) for helminth diagnosis.

### Study area

The Manhiça District covers an area of 2,380 km^2^ and has an estimated population of approximately 209,000 inhabitants [32,33]. The district is predominantly rural to semi-rural, with agricultural activities as the main source of income [34]. Malaria transmission is perennial, with peaks occurring during the rainy season from November to April. Malaria remains one of the leading causes of fever in the district, although its incidence has declined over time [35,36]. In Mozambique, helminth infections are widely distributed, with a reported STH prevalence of approximately 13% in Manhiça [37,38]. The district has one main referral facility, the Manhiça District Hospital, which receives patients from peripheral health centres as well as from surrounding areas. The Ilha Josina Machel Health Centre is one such peripheral facility, serving approximately 8,288 inhabitants in the Ilha Josina area [34].

### Helminth and *P. falciparum* detection

STH were identified microscopically using Telemann’s sedimentation/concentration technique [39], and remaining stool aliquots were frozen at –80°C for subsequent multi-parallel quantitative polymerase chain reaction (qPCR) analysis [40,41]. After thawing, DNA was isolated from 50 mg of stool using the MP FastDNA for Soil Kit (MP Biomedicals, Solon, OH, USA). Each qPCR was carried out in duplicate in a 7 µL reaction mixture comprising 3.5 µL of TaqMan Fast Advanced Master Mix (Applied Biosystems), 2 µL of extracted DNA, and 1.5 µL of species-specific primer/probe sets labelled with FAM and a minor groove binder. Amplification was performed on an ABI 7500 Fast Real-Time PCR instrument (Applied Biosystems). Samples with cycle threshold (Ct) values greater than 38 were classified as negative. This cutoff was defined based on the detection limits of each assay, established using serial dilutions of parasite-specific plasmid controls. The Ct value of 38 corresponded to the lower limit of the standard curves, beyond which further dilutions were not detectable [41].

*P. falciparum* parasites were detected by Giemsa-stained thick and thin smears, with quantification based on microscopy [42]. Blood smears were observed independently by two trained microscopists, and a third independent reading was conducted to resolve discrepancies. Samples negative by microscopy underwent duplicate testing using a real-time qPCR assay targeting the 18S rRNA gene [43], following DNA extraction from DBS using the QIAamp DNA Mini kit (QIAGEN, Hilden, Germany). Extraction blanks and negative controls were included in each qPCR plate. Parasite density was derived from standard curves constructed with *P. falciparum*-positive controls of known parasitemia. Samples with no amplification were considered negative.

### Cytokine, chemokine, and growth factor profiling

Plasma concentrations (pg/mL) of 30 immune mediators were determined using the Human Magnetic Cytokine 30-Plex Panel (Life Technologies), according to the manufacturer’s instructions. Analytes in the panel included granulocyte colony-stimulating factor (G-CSF), granulocyte-macrophage colony-stimulating factor (GM-CSF), interferon (IFN)-α, IFN-γ, IL-1β, IL-1RA, IL-2, IL-2R, IL-4, IL-5, IL-6, IL-7, IL-8, IL-10, IL-12 (p40/p70), IL-13, IL-15, IL-17, tumor necrosis factor (TNF), eotaxin, IFN-γ-induced protein (IP-10), monocyte chemoattractant protein (MCP-1), monokine induced by IFN-γ (MIG), macrophage inflammatory protein (MIP)-1α, MIP-1β, RANTES (regulated on activation, normal T-cell expressed and secreted), epidermal growth factor (EGF), fibroblast growth factor (FGF), hepatocyte growth factor (HGF), and vascular endothelial growth factor (VEGF). The protocol was modified by reducing the volume of plasma, standards, and reagents by half, which did not compromise assay performance [44]. Analyte concentrations were estimated by fitting median fluorescence intensity (MFI) values to a four- or five-parameter logistic regression model using the drLumi R package [45]. Standard curves were generated from 16 two-fold serial dilutions of manufacturer-provided reference material with known concentrations. Values falling below the limit of detection were replaced with half the lower limit of quantification (LLOQ). For all samples, RANTES results clustered close to the upper limit of quantification (ULOQ) and were therefore removed from the analyses since they were not informative. For the remaining cytokines, IL-17 showed the highest proportion of samples below the LLOQ (30%). We chose to retain IL-17 because the majority of samples yielded quantifiable values and the analyte displayed consistent patterns in the heatmap analysis; the samples below the LLOQ were imputed by assigning half of the LLOQ (LLOQ/2). Acquisition was carried out on the Luminex 100/200 platform using the Xponent software.

### Antibody quantification

Antibody responses were characterised against nine STH antigens, Na-GST-1 (*N. americanus* glutathione S-transferase-1), Na-APR-1 (*N. americanus* aspartic protease-1), Na-SAA-2 (*N. americanus* surface-associated antigen-2), Ay-CP2 (*A. ceylanicum* cysteine protease-2), Tm-WAP (*T. muris* whey acidic protein), Tm-16 (*T. muris* 16-kDa excretory/secretory antigen), As-16 (*A. suum* 16-kDa secreted antigen), As-37 (*A. suum* 37-kDa immunodominant antigen), and NIE (a recombinant *S. stercoralis* L3 diagnostic antigen), as well as two *Schistosoma spp*. antigens, Sm-SERPIN (*S. mansoni* serine protease inhibitor) and Sh-SERPIN (*Schsitosoma haematobium* serine protease inhibitor) (S1 Table). NIE was expressed in *Escherichia coli* by Sukwan Handali (Centers for Disease Control and Prevention, USA) using a plasmid construct provided by Thomas Nutman (National Institutes of Health and National Institute of Allergy and Infectious Diseases, USA). The antigens As16, As37, AyCp2, NaAPR1, NaGST1, NaSAA2, Tm16, and TmWAP were produced using *Pichia pastoris* X-33 yeast cultures at Baylor University (USA).

Quantitative suspension array technology (qSAT) was used on the xMAP Luminex platform to measure antigen-specific IgG, IgG subclasses (IgG1–4), IgA, IgM, IgE, and total IgE [46]. Antigens were covalently bound to MAGPLEX carboxylated microspheres (6.5 μm; Luminex Corporation, Austin, USA) following previously published protocols [46] and bead coupling was verified prior to antibody detection assays. Plasma samples were tested at different final dilutions depending on the antibody class or subclass: 1/1000 for IgG and IgM, 1/250 for IgG1 and IgA, 1/100 for IgG2–4, 1/20 for IgE, and 1/50 for total IgE. Multiplex assays were carried out with 1500 beads per analyte per well, while total IgE was quantified in singleplex using 3000 beads per well. To minimise IgG interference, samples analysed for IgA, IgM, and IgE were pre-incubated with the IgG-blocking reagent GullSORB at a 1:10 ratio (sample to GullSORB). Samples were also randomised prior to assay setup to minimise plate variability.

Magnetic microspheres coupled to either specific antigens or capture anti-human IgE monoclonal antibodies (Abcam Limited) were incubated with plasma samples for one hour at room temperature under agitation (900 rpm). Reactions were carried out in μClear flat-bottom plates (Greiner Bio-One): 96-well plates for antigen-specific IgE and 384-well plates for IgG, IgG subclasses, IgA, IgM, and total IgE. Plates were washed three times with 100 or 200 µL/well, depending on the plate, of PBS containing 0.05% Tween-20 (PBS-T) using an automated washer (Agilent BioTek, USA), followed by isotype-specific detection steps. For IgG, IgA, IgM, and IgE, secondary detection involved PE-conjugated antibodies: anti-human IgG (Moss Bio, 1/400), anti-human IgA (Moss Bio, 1/100), anti-human IgM (Moss Bio, 1/300), and anti-human IgE (SouthernBiotech, 1/100). For IgG1, IgG3, and total IgE, biotinylated detection antibodies used were [anti-human IgG1 (ThermoFisher, 1/2000), anti-human IgG3 (ThermoFisher, 1/250), anti-human IgE (Moss Bio, 1/100)], followed by Streptavidin-R-Phycoerythrin (Moss Bio, 1/500). IgG2 and IgG4 were detected with mouse anti-human IgG2 (ThermoFisher, 1/125) or IgG4 (ThermoFisher, 1/125), followed by biotinylated anti-mouse IgG (Moss Bio; 1/500 and 1/250, respectively), and subsequent incubation with Streptavidin-R-Phycoerythrin. All incubations were performed for 30 min at room temperature with shaking at 900 rpm in the dark, with washes between steps. Beads were finally resuspended in Luminex buffer (PBS supplemented with 1% BSA, 0.05% Tween-20, and 0.05% sodium azide) prior to acquisition. Beads were acquired on a FlexMap 3D system, with a minimum of 50 beads per analyte, and data were reported as MFI.

Antibody assays incorporated positive controls, negative controls, and blanks to ensure quality control. For IgG and IgG subclass quantification, the positive control consisted of the WHO reference reagent (Human serum 10/198, NIBSC, UK), which was tested in 12 two-fold serial dilutions starting at 1/100. For IgA and IgM quantification, the positive control consisted of a plasma pool of positive samples from earlier studies, also prepared in 12 two-fold serial dilutions from 1/100. For the quantification of antigen-specific IgE, a previously characterised study sample served as the positive control, assayed in 8 two-fold serial dilutions starting at 1/20. For total IgE quantification, the positive control consisted of the WHO IgE reference standard (Third International Standard, human serum 11/234, NIBSC, UK) with 12 two-fold dilutions beginning at 1/50. Each assay also included 10 negative control plasma samples (8 for antigen-specific IgE) obtained from unexposed adults, which were tested at the same dilutions as the study samples and distributed across the plates. Two blanks containing only Luminex buffer were run per plate to assess background fluorescence.

### Antibody data pre-processing

Quality control was performed by examining positive control curves, the distribution of test samples, the blank background signal, and the unspecific signal of negative controls within and across assay plates. No inter-plate discrepancies were observed for IgG, IgG subclasses (IgG1–4), IgA, IgM, or total IgE. However, a plate effect was detected for IgE in two of the six plates (plates 2 and 5). To correct for this, normalisation was carried out using the control sample results. Specifically, the median MFI per analyte from the controls of the four unaffected plates was divided by the median MFI per analyte from the controls in plates 2 and 5 to generate a normalisation factor. All MFI values from plates 2 and 5 were then adjusted by multiplying them by their respective normalisation factor.

### Statistical analysis

Participant infection status was assigned using the following algorithm. For *P. falciparum*, children were classified as infected if they were positive by microscopy or, in the case of microscopy-negative samples, positive by qPCR. For helminths, children were classified as infected if any diagnostic method yielded a positive result. For both infections, children were classified as uninfected if all diagnostic methods were negative. For analyses, participants were grouped into two categories: helminth-infected or malaria and helminth coinfected. Continuous variables were compared using the Wilcoxon rank-sum test, while categorical variables were evaluated with Pearson’s χ² or Fisher’s exact test. Not all clinical variables were available for all participants due to incomplete data in clinical forms, therefore, analyses were performed based on available data. Anaemia was defined as hematocrit < 33% [47,48]. Cytokine, chemokine, growth factor, and antibody values were log10-transformed prior to analysis. Hierarchical clustering heatmaps (Euclidean distance, complete linkage) were generated to visualise immune patterns, taking into account factors such as infection group, age, sex, site, and anaemia.

Linear regression models were applied to: (i) explore associations between demographic/clinical factors and immune responses, (ii) evaluate the relationship of coinfection with cytokine and antibody levels, and (iii) evaluate correlations between antibody and cytokine responses. Results are presented as percentage changes with 95% confidence intervals (CI). In regression models where both predictors and outcomes were log10-transformed (log–log models), β coefficients were converted to percentage changes using the formula ((10^(β*log10(1.1)))-1)*100. This expression corresponds to the percentage variation in the outcome associated with a 10% increase in the predictor. For models in which only the outcome was transformed (log–linear models), coefficients were expressed as ((10^beta)-1)*100, reflecting the percentage change in the dependent variable for a one-unit increase in a continuous predictor or relative to the reference group for categorical predictors. Multivariable linear regression models were adjusted for potential confounders (including age, sex, and area of residence) based on prior knowledge and directed acyclic graph (DAG) informed confounder assessment. To account for multiple comparisons, the Benjamini–Hochberg method was applied, with p < 0.05 considered significant. However, given the exploratory nature of this study and the sample size, biologically plausible findings were also discussed, even if they did not meet strict multiple testing thresholds, thus, both adjusted and unadjusted p-values are presented in the plots.

Effect modification of the associations of antibody responses with cytokine concentrations by infection group was examined by including interaction terms in regression models and comparing nested models using ANOVA. Stratified analyses were performed when significant interactions were detected.

Partial Least Squares Discriminant Analysis (PLS-DA) was conducted using the mixOmics R package [49] to assess multivariate patterns of immune markers in relation to infection status.

All analyses were performed in R version 4.4.0 with the following packages: tidyverse [50], gtsummary [51], ggpubr [52], ggbeeswarm [53], ComplexUpset [54], and pheatmap [55].

## Results

### Parasitological, demographic, and clinical characteristics of the study population

**Fig 1:**
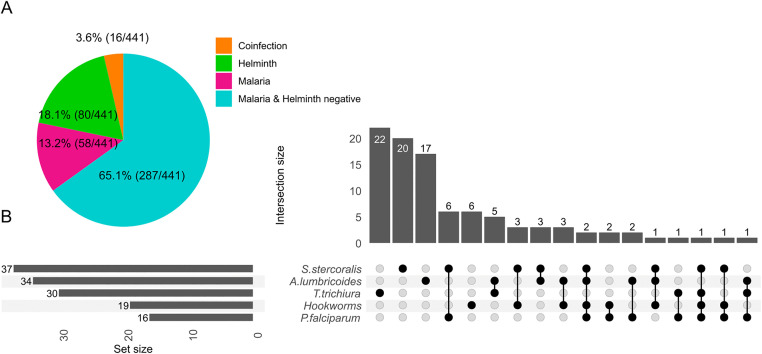
Helminth and *Plasmodium falciparum* prevalence in the study population. **(A)** Pie chart showing the distribution of study participants by infection status (N = 441). **(B)** UpSet plot showing helminth and *P. falciparum* infection prevalence and overlap among helminth-positive study participants (N = 96). The center matrix illustrates the existing parasite intersections in single infection and coinfection. The vertical barplot indicates the number of individuals at each intersection, indicating single or multiple infections. The horizontal barplot represents the number of individuals positive for each parasite.

Of the 441 recruited participants, 58 (13.2%) had only malaria, 80 (18.1%) had only helminths, and 16 (3.6%) had both infections (Fig 1A). The remaining 287 (65.1%) participants were negative for malaria and the screened STHs, but also presented with diverse symptoms and were clinically diagnosed with other diseases (S1 Fig). Of the STH infections, *S. stercoralis* was the most prevalent (39%, 37/96), followed by *A. lumbricoides* (35%, 34/96), *T. trichiura* (31%, 30/96), and hookworms (*A. duodenale* and *N. americanus*) (20%, 19/96) as reflected in Table 1 and Fig 1B.

**Table 1:** Parasitological, demographic, and clinical characteristics of the study participants.

Characteristic	N	Overall N = 96^1^	Coinfection N = 16^1^	Helminth N = 80^1^	*p-*value
Age group	96				0.064^2^
2–5 yr		31 (32%)	2 (13%)	29 (36%)	
6–10 yr		65 (68%)	14 (88%)	51 (64%)	
Area	96				0.025^2^
Ilha Josina		31 (32%)	9 (56%)	22 (28%)	
Manhiça-sede		65 (68%)	7 (44%)	58 (73%)	
Sex	96				>0.9^2^
Female		42 (44%)	7 (44%)	35 (44%)	
Male		54 (56%)	9 (56%)	45 (56%)	
HIV	96	3 (3.1%)	0 (0%)	3 (3.8%)	>0.9^3^
Anaemia (hematocrit <33%)	89	9 (10%)	5 (33%)	4 (5.4%)	0.006^3^
Hematocrit (%)	89	38.0 (35.0, 40.0)	37.0 (32.0, 40.5)	38.0 (35.0, 40.0)	0.4^4^
Fever (yes/no)	87	55 (63%)	12 (75%)	43 (61%)	0.3^2^
Temperature	87	36.50 (36.25, 37.10)	36.55 (36.18, 37.88)	36.50 (36.30, 37.10)	0.7^4^
Cough (yes/no)	87	40 (46%)	8 (50%)	32 (45%)	0.7^2^
Diarrhea (yes/no)	87	1 (1.1%)	0 (0%)	1 (1.4%)	>0.9^3^
Vomit (yes/no)	87	5 (5.7%)	2 (13%)	3 (4.2%)	0.2^3^
Wheezing (yes/no)	87	2 (2.3%)	1 (6.3%)	1 (1.4%)	0.3^3^
**Parasites**					
Hookworms (*A. duodenale, N. americanus*)	96	19 (20%)	6 (38%)	13 (16%)	0.081^3^
*A. lumbricoides*	96	34 (35%)	5 (31%)	29 (36%)	0.7^2^
*T. trichiura*	96	30 (31%)	3 (19%)	27 (34%)	0.2^2^
*S. stercoralis*	96	37 (39%)	10 (63%)	27 (34%)	0.031^2^
Number of helminth infections	96				0.020^3^
1		76 (79%)	11 (69%)	65 (81%)	
2		16 (17%)	2 (13%)	14 (18%)	
3		4 (4.2%)	3 (19%)	1 (1.3%)	

^1^n (%); Median (IQR), ^2^Pearson’s Chi-squared test, ^3^Fisher’s exact test, ^4^Wilcoxon rank sum test.

The subsequent analysis included the 96 participants diagnosed with helminth infections. Of these, 68% (n = 65) were aged 6–10 years, and the same proportion (68%, n = 65) resided in the semi-urban area of Manhiça-sede, which had a statistically significantly higher proportion of only-helminth individuals compared with Ilha Josina Machel (p = 0.025, Table 1). Overall, 44% (n = 42) of the participants were female, and HIV prevalence was 3.1% (n = 3), with all positive cases occurring in the helminth-only group (Table 1).

The prevalence of anaemia (hematocrit <33%) was 10% (9/89) overall but significantly higher in proportion among coinfected individuals (33%, 5/15) compared to those with helminth infection alone (5.4%, 4/74) (p = 0.006), whereas median hematocrit levels were similar across both groups (Table 1). The most prevalent symptoms were fever (63%, 55/87), with a median body temperature of 36.5°C (IQR: 36.25–37.10), and cough (46%, 40/87) (Table 1 and S1 Fig). Other clinical symptoms, such as vomiting (5.7%, 5/87), wheezing (2.3%, 2/87), and diarrhoea (1.1%, 1/87), were also reported (Table 1 and S1 Fig). No statistically significant differences were found in age, sex distribution, HIV status, and clinical symptoms between infection groups.

In terms of hospital-based diagnoses, the coinfected participants were mainly diagnosed with malaria, with no reference to the concurrent helminth infection (S1 Fig). Children with helminth monoinfection were mainly diagnosed with respiratory infections and general symptoms and signs, which include fever of unknown origin, hypothermia, dry mouth, and other causes of morbidity (S1 Fig).

### Association of demographic and clinical variables with plasma cytokine concentrations

**Fig 2:**
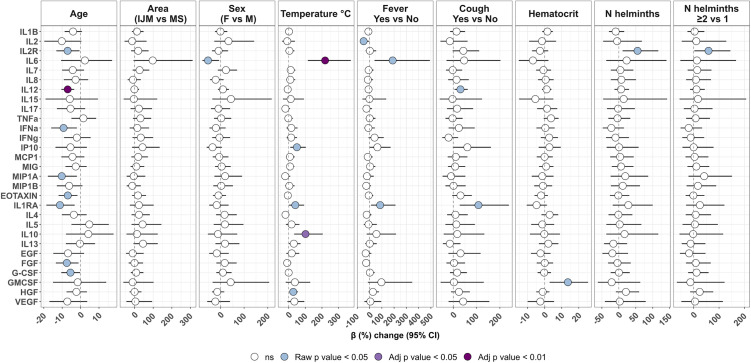
Association of demographic and clinical variables with plasma cytokine concentrations. Forest plots depict the association of circulating cytokine concentrations (log10 pg/mL) with age (N = 96), area (N = 96), sex (N = 96), temperature (N = 87), fever (N = 87), cough (N = 87), hematocrit percentage (N = 89), and the number of infecting helminth (N helminths) species in both continuous and categorical forms (N = 96). The estimates (points) and 95% confidence intervals (CIs) were obtained from univariable linear regression models and transformed into percentages for interpretation purposes (see the statistical analysis section for details). The colour of the points in the forest plots reflects the significance of the p-values before and after adjustment for multiple testing using the Benjamini-Hochberg method: empty circles indicate non-significant results (ns), light blue denotes a raw p-value < 0.05, light purple indicates an adjusted p-value < 0.05, and dark purple an adjusted p-value < 0.01.

Overall, plasma cytokine concentrations were not associated with particular demographic or clinical variables in univariable linear regression models (Fig 2). Exceptions included age, which was negatively associated with IL-12 concentrations (β = −0.032, 95% CI: −0.048 to −0.015, adjusted p = 0.006), and body temperature, which was positively associated with the pro-inflammatory cytokine IL-6 (β = 0.503, 95% CI: 0.332 to 0.675, adjusted p = 0.0001) and the regulatory cytokine IL-10 (β = 0.303, 95% CI: 0.123 to 0.484, adjusted p = 0.018).

### Association of demographic and clinical variables with antibody responses

**Fig 3:**
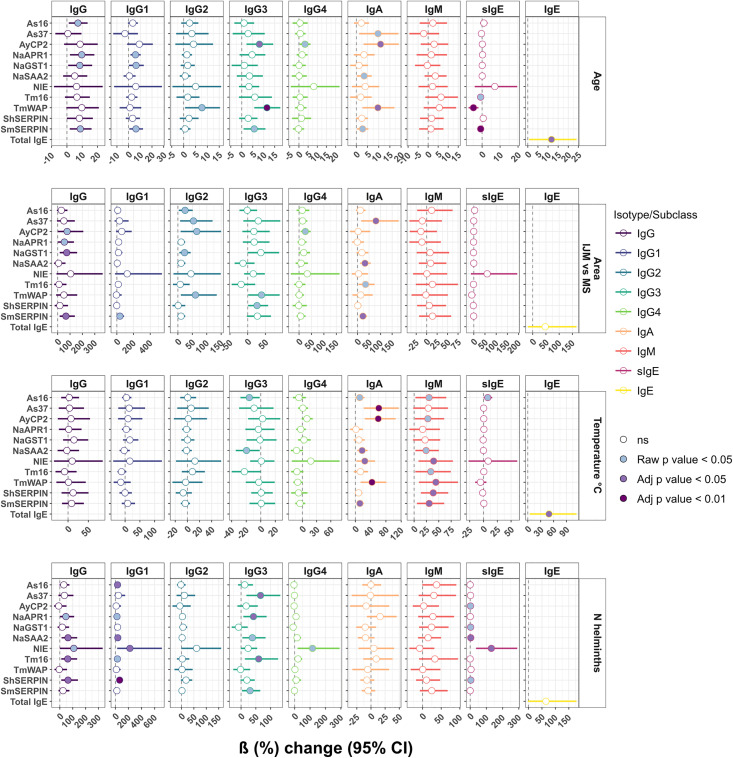
Association of demographic and clinical variables with antibody levels. Forest plots illustrate the association of antibody levels (log10 MFI) with age, area of residence, body temperature and number of infecting helminth species. The estimates (points) and 95% confidence intervals (CIs) were obtained from univariable linear regression models and transformed into percentages for interpretation purposes (see the Statistical Analysis section for details). The colour of the points in the forest plots reflects the significance of the p-values before and after adjustment for multiple testing using the Benjamini-Hochberg method: empty circles indicate non-significant results (ns), light blue denotes a raw p-value < 0.05, light purple indicates an adjusted p-value < 0.05, and dark purple an adjusted p-value < 0.01. The colour of the lines represents the antibody isotype or subclass. N = 96, with the exception of body temperature, where the N was 87.

Age was significantly associated with multiple helminth-specific antibody responses (Fig 3). Specifically, IgG3 and IgA responses to Tm-WAP and Ay-CP2, as well as total IgE, increased with age, whereas IgE responses to Tm-WAP and Sm-SERPIN decreased with age. Residing in Ilha Josina Machel compared to Manhiça Sede was associated with an increase in IgG responses to Na-GST-1 and Sm-SERPIN, as well as IgA responses to As-37, Na-SAA-2, and Sm-SERPIN (Fig 3). An increase in body temperature was significantly associated with an increase in total IgE, IgA responses against Ay-CP2, As-37, Tm-WAP, Na-SAA-2, Sm-SERPIN and NIE, as well as IgM levels against NIE, Tm-WAP, Sh-SERPIN, and Sm-SERPIN (Fig 3). The presence of fever was also associated with an increase in IgG4-Ay-CP2 levels (adjusted p = 0.034) (S2 Fig). Antibody responses showed a trend towards a positive association with the number of helminth species infecting each individual. Specifically, this association was observed for IgG responses to Na-SAA-2, Sh-SERPIN, and Tm-16, for the cytophilic antibodies IgG1 and IgG3, with IgG1 responses to As-16, Na-SAA-2, NIE, and Sh-SERPIN, and IgG3 responses to As-37, Na-APR-1, and Tm-16, and for IgE responses to Na-SAA-2 and NIE (Fig 3). Sex, cough status, and hematocrit percentage were not associated with any of the measured antibody responses (S2 Fig).

### *P. falciparum* coinfection is associated with increased circulating cytokine responses in helminth-infected children

**Fig 4:**
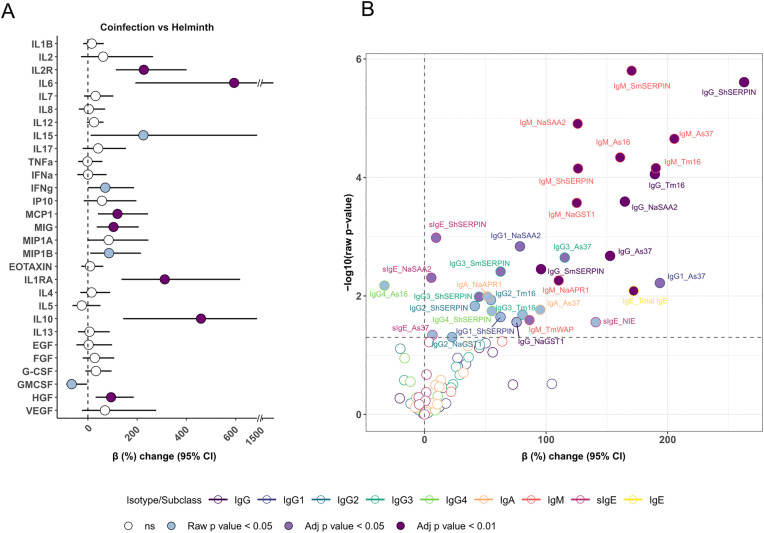
Effect of *Plasmodium falciparum* coinfection on (A) cytokine, chemokine, growth factors, and (B) helminth-specific antibody responses, among helminth-positive participants. Multivariable linear regression models (adjusted by age, sex, and area of residence) were used, and (A) forest plots illustrate obtained estimates (points) and 95% confidence intervals (CIs) and (B) volcano plots illustrate only the obtained estimates (points) transformed into percentages for interpretation purposes (Log-linear transformed: see the Statistical Analysis section for details). The colour of the circle borders represents the antibody isotype or subclass. The colour of the points in the forest plots reflects the significance of the p-values before and after adjustment for multiple testing using the Benjamini-Hochberg method: empty circles indicate non-significant results (ns), light blue denotes a raw p-value < 0.05, light purple indicates an adjusted p-value < 0.05, and dark purple an adjusted p-value < 0.01. The x-axis incorporates breaks to accommodate wide confidence interval ranges. The colours of the circle borders represent the antibody isotype or subclass (B). N = 96.

In multivariable linear regression models, we observed a trend of elevated cytokine responses among children coinfected with helminths and *P. falciparum* compared to children who were positive only for helminths. Specifically, coinfected participants had increased concentrations of pro-inflammatory cytokines IL-2R and IL-6, the cell-recruiting chemokines MCP-1 and MIG, the anti-inflammatory proteins IL-1RA and IL-10, as well as the growth factor HGF (β estimate percentage changes ranged from 94% to 594%, adjusted p < 0.01) (Figs 4A and S3).

### *P. falciparum* coinfection is associated with increased helminth-specific antibody levels

Antibody response profiles in the study population, using hierarchical clustering, showed heterogeneity across antigen-specific responses and antibody isotypes, with subtle clustering patterns by infection group (S4 Fig). After adjusting for relevant covariates (age, sex, and area of residence), coinfection with *P. falciparum* was associated with significantly increased antibody responses in multivariable linear regression models, with this trend being more pronounced for helminth-specific IgG and IgM responses (Figs 4B, S5 and S7–S16). IgM responses were elevated against As-16, As-37, Na-APR-1, Na-GST-1, Na-SAA-2, Sh-SERPIN, Sm-SERPIN, Tm-16, and Tm-WAP (β-estimate percentage changes varied from 86% to 205%, adjusted p < 0.031). Total IgG responses were significantly higher against As-37, Na-SAA-2, Sh-SERPIN, Sm-SERPIN and Tm-16, with β-estimate percentage changes ranging from 93% to 263% (adjusted p < 0.01). Consistently, IgG subclass responses were generally elevated in coinfected children, with increases observed for the cytophilic subclasses IgG1 (As-37, Na-SAA-2) and IgG3 (As-37, Sm-SERPIN, Sh-SERPIN), with β-estimate percentage changes ranging from 45% to 194% (adjusted p < 0.05).

Increased levels were also observed for antigen-specific IgE, including Sh-SERPIN (β = 9.53, 95% CI: 3.84–15.54, adjusted p = 0.011), Na-SAA-2 (β = 5.6, 95% CI: 1.71–9.63, adjusted p < 0.027), as well as increased total IgE (β = 172.05, 95% CI: 30.37–467.68, adjusted p = 0.008). Notably, although helminth-specific IgE responses were significantly increased, the β estimates were generally lower compared to those of other isotypes.

Interestingly, several antigens, including Sh-SERPIN, Sm-SERPIN, As-37, Na-SAA-2, Na-GST-1, and Tm-16, were repeatedly associated with increased responses across multiple isotypes (IgG, IgG subclasses, IgM, and IgE) among coinfected children.

### Concurrent *P. falciparum* infection modifies the impact of cytokines on helminth-specific antibody responses

**Fig 5:**
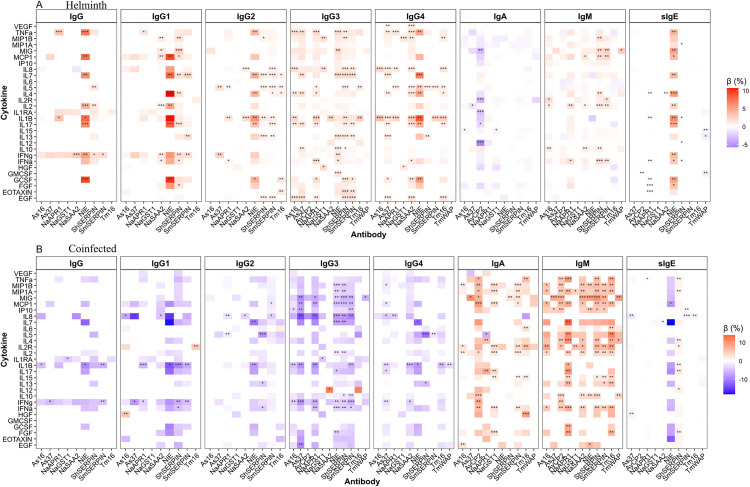
Effect of cytokine concentrations on helminth-specific antibody responses stratified by group. Heatmaps illustrate the association of cytokine concentrations (log10 pg/mL) with antibody responses (log10 MFI) in **(A)** helminth-positive and **(B)** *P. falciparum* coinfected study participants. Associations were performed using multivariable linear regression models, and the obtained estimates (colours) illustrated in the plots were transformed into percentages for interpretation purposes (Log-log transformed: see the Statistical Analysis section for details). The asterisks in the plots reflect the significance of the p-values for each antibody-cytokine association before and after adjustment for multiple testing using the Benjamini-Hochberg method: * raw p-value < 0.05, ** adjusted p-value < 0.05, and *** adjusted p-value < 0.01. The plots only show antibody-cytokine pairs where the model including the interaction term provided a significantly better fit than the model without interaction, as determined by ANOVA (raw p < 0.05), thus showing that the relationship between antibody and cytokine levels differed between study groups for these pairs. N = 96.

**Fig 6:**
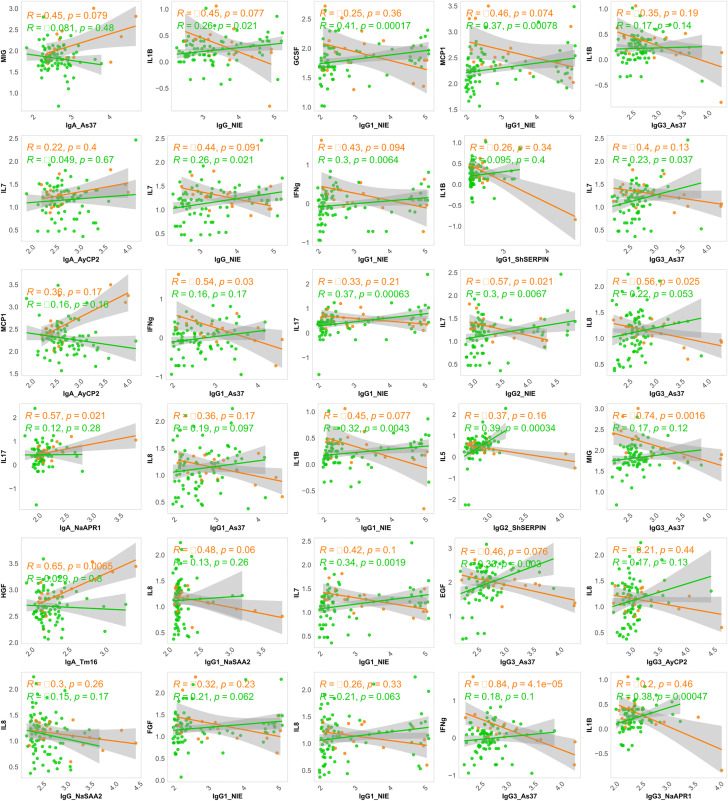
Association of cytokine concentrations with antibody levels. Scatter plots show correlations between antibody (log10 MFI) and cytokine levels (log10 pg/mL) stratified by infection group. Helminth mono-infected individuals are shown in green, and coinfected individuals are shown in orange. Spearman correlation coefficients and p-values are shown in each plot. A linear regression line with a 95% confidence interval is included. The scatter plots continue in S6 Fig and S6 Fig (cont).

The association between cytokine concentrations and antibody levels was evaluated using multivariable linear regression models that included an interaction term for *P. falciparum* infection status, allowing us to assess whether cytokine–antibody relationships differed between helminth-only and coinfected children. Assessment of the association between cytokine concentrations and antibody levels revealed that the effect of cytokines on antibody responses appeared to be altered by concurrent *P. falciparum* infection (Figs 5, 6 and S6). Among children positive only for helminths, cytokine concentrations were positively associated with helminth-specific IgG, IgG1-4, and IgE levels, with particularly strong positive associations observed for antibody responses against NIE (Figs 5A, 6 and S6). In contrast, among children coinfected with helminths and *P. falciparum*, an opposing trend was observed, whereby cytokine concentrations were negatively associated with helminth-specific IgG, IgG1-4, and IgE levels, also with particularly strong negative associations for antibodies against NIE (Figs 5B, 6 and S6). Opposing patterns were also observed for helminth-specific IgA, where cytokine concentrations were mainly negatively associated in the helminth group (Figs 5A, 6 and S6) and positively associated in the coinfected group (Figs 5B, 6 and S6), with this trend being particularly noticeable for responses against Ay-CP2.

### Multimarker analysis of the effect of malaria coinfection on helminth responses

**Fig 7:**
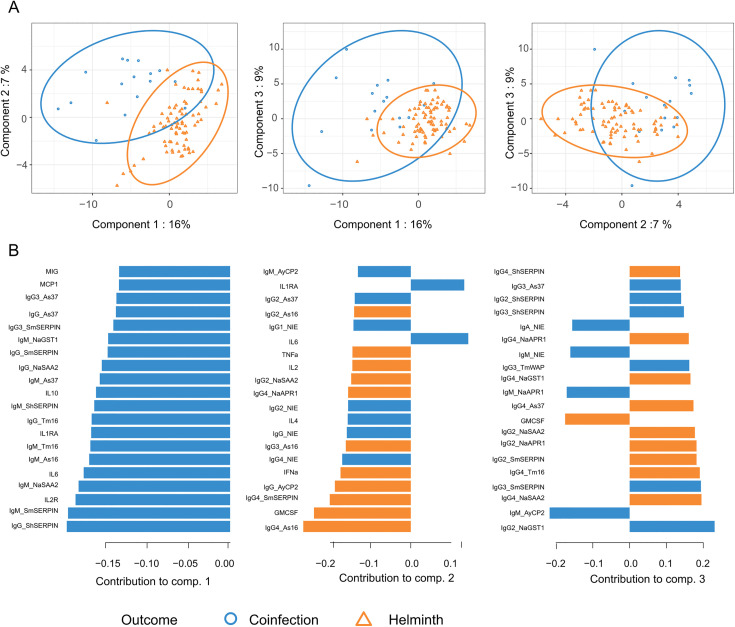
Partial Least-Squares Discriminant Analysis (PLS-DA) of antibody and cytokine responses. **(A)** Score plots show the distribution of individuals by infection groups (outcome). The first three principal components, which explain the highest percentage of variance in the immune responses, are represented. **(B)** Bar plots illustrate the first 20 variables that contribute to the principal components.

To further assess the impact of concurrent *P. falciparum* infection on helminth responses, a PLS-DA machine learning analysis was performed. This multimarker analysis revealed clear clustering of the two infection groups, indicating distinct immune patterns between coinfected and helminth-only children (Fig 7A). In component 1, the separation of the coinfected group was primarily driven by antibody and cytokine responses elevated in coinfected children (Fig 7B). In component 2, immune markers that were reduced in coinfected participants contributed more strongly to the separation of helminth-only participants (Fig 7B).

## Discussion

Our main results suggest that malaria coinfection significantly alters helminth-associated immunity, as evidenced by the amplified cytokine and antibody responses, as well as the modified cytokine-antibody relationships in coinfected children compared to those diagnosed with helminths only. Importantly, our findings also suggest that *P. falciparum* and helminth coinfection may have a significant impact on disease morbidity, as indicated by the higher proportion of anaemic children in the coinfected group.

In agreement with a previous study [56], we observed that the coinfected participants had higher levels of pro-inflammatory cytokines, accompanied by increased expression of anti-inflammatory cytokines, when compared to the group of children positive only for helminths. The cytokine profile observed in these coinfected participants is consistent with the immune responses seen in acute malaria, where a pro-inflammatory response is elicited to limit parasite proliferation, followed by an anti-inflammatory and regulatory response, including the production of IL-10, to control excessive inflammation and immunopathology, although it can also contribute to sustained parasite survival [57,58]. This suggests that the cytokine profile observed in these symptomatic coinfected children, particularly the upregulation of pro-inflammatory cytokines such as IL-2R and IL-6, is likely driven mainly by the acute malaria infection. Furthermore, the increased expression of IL-10 in the coinfected children may be a synergistic effect resulting from the concurrent presence of *P. falciparum* and helminths, as both are inducers of anti-inflammatory and regulatory responses, eventually [59,60].

As observed in our previous study [31] and earlier reports [25,26], coinfection was also associated here with elevated helminth-specific antibody levels across most measured isotypes and subclasses. We also found that while in helminth monoinfected individuals, the associations between cytokines and antibody responses were mainly positive, these turned out either negative or non-correlated in the coinfected group, with the exception of IgA and IgM responses. At first glance, this may seem inconsistent, since both cytokine and IgG levels are higher in the coinfected group. However, these findings are not necessarily mutually exclusive. One possibility is that the higher antibody levels observed in coinfected individuals are due to polyclonal B cell activation, which induces a global elevation of antibody levels [61–63] without necessarily a direct association with cytokine concentrations. Another possible explanation is non-linear relationships between cytokine and antibody levels [64] or a dysregulation of the coordination between cytokine signalling and antibody production once a certain level of immune activation is reached [65]. In addition, we might consider that plasma cytokines reflect systemic inflammation, whereas antibody production may depend on local signalling in the germinal centres [66,67]. Furthermore, multimarker analyses suggested that the coinfected and helminth-only children likely had distinct immune responses. Together, these findings highlight the potential of malaria to alter helminth-induced immunity.

Our results also suggest that concurrent infections could have an impact on host clinical outcomes. We showed that the prevalence of anaemia was significantly higher among children coinfected with *P. falciparum* and helminths than among those diagnosed solely with helminths. This aligns with previous studies demonstrating that coinfection with malaria and helminths, particularly hookworms and *Schistosoma* species, can synergistically exacerbate anaemia through mechanisms such as red blood cell destruction, cytokine-induced dyserythropoiesis, and chronic intestinal blood loss [68–70].

This study had some limitations. First, participants in the helminth-only group may have had undiagnosed additional infections that contributed to their symptoms and hospital visit, potentially influencing the results of our immune markers. Furthermore, as participants were recruited from a hospital setting, the study population may not be fully representative of children in the broader community, which may limit the generalizability of our findings. Missing clinical variables for some participants could have influenced analysis outcomes where these variables were included. Furthermore, we did not formally adjust for confounding in anaemia analysis; therefore, we cannot exclude confounding from unmeasured factors such as nutritional status. In addition, we did not have data on helminth burden, therefore, we cannot exclude the possibility that this factor may have influenced the observed associations. This study also did not account for seasonal variation in malaria and helminth transmission, and given the known seasonality of malaria in particular, this may have influenced our findings. The small number of coinfected participants compared to those infected solely with helminths may have reduced the statistical power to detect additional associations. Additionally, the sample size prevented stratified analyses by helminth species, which could have masked species-specific effects. Finally, the cross-sectional study design limits the ability to infer causal relationships. However, despite these constraints, the study provides mechanistic insights into the potential impact of malaria coinfection on helminth infections, based on a comprehensive panel of helminth antigens and cytokines. Specifically, the added value of this study lies in the combined analysis of clinical data, antibody (including antigen-specific IgE) and cytokine responses, whereas many previous studies have examined these components in isolation. Furthermore, our study includes a broad panel of helminth antigens alongside a wide range of cytokines, enabling the simultaneous analysis of multiple parameters within the same population. Lastly, while much of the existing literature on malaria-helminth coinfections has focused on the effect of helminths on malaria, the inverse relationship has received comparatively less attention.

Taken together, our findings suggest that concurrent malaria and helminth infections may potentially exacerbate helminth clinical outcomes as well as modulate helminth-specific immune responses, potentially influencing parasite expulsion or survival. Future studies should further evaluate the quality (e.g., avidity, Fc glycosylation) and functional activity of anti-helminth antibodies induced in both study groups to elucidate whether coinfection could affect worm expulsion and enhance or impair anti-helminth vaccine responses.

## Supporting information

S1 Table Helminth antigens. (DOCX)

S1 Fig Clinical symptoms and diagnoses stratified by infection status. The barplots show the frequency in percentage of (A) reported symptoms and (B) hospital-based diagnosis.
(TIF)

S2 Fig Association of demographic and clinical variables with antibody levels. Forest plots illustrate the association between antibody levels (log10 MFI) and sex (N = 96), fever (N = 87), cough (N = 87), hematocrit percentage (N = 89), and having more than two infecting helminth species (N = 96). The estimates (points) and 95% confidence intervals (CIs) were obtained from univariable linear regression models and transformed into percentages for interpretation purposes (see the Statistical Analysis section for details). The colour of the points in the forest plots reflects the significance of the p-values before and after adjustment for multiple testing using the Benjamini-Hochberg method: empty circles indicate non-significant results (ns), light blue denotes a raw p-value < 0.05, light purple indicates an adjusted p-value < 0.05, and dark purple an adjusted p-value < 0.01. The colour of the lines represents the antibody isotype or subclass.
(TIF)

S3 Fig Hierarchical clustering heatmap of circulating cytokine responses. Columns represent study participants, and rows represent plasma cytokine concentrations (log10 pg/mL), which are shown on a colour scale from yellow to orange. On top of the heatmap, infection status, sex, age group, area of residence, and anaemia are shown in relation to cytokine concentrations.
(TIF)

S4 Fig Hierarchical clustering heatmap of antibody responses. Heatmaps show an overview of antibody (IgG, IgG1-4, IgA, IgM, IgE, and total IgE) responses against helminth antigens. Columns represent study participants, and rows represent antigen-specific antibody levels. Antibody levels (Log10 MFI) are shown on a colour scale from yellow to orange. On top of the heatmap, infection status, sex, age group, area of residence, anaemia, and specific helminth infection status are shown in relation to antibody levels.
(TIFF)

S5 Fig Association of coinfection with antibody responses. A multivariable linear regression model (adjusted by age, sex, and area) was performed to assess the potential effect of coinfection with malaria and helminths on antibody responses (log10 MFI), considering these possible confounding factors. Forest plots illustrate estimates (points) and 95% confidence intervals (CIs) transformed into percentages for interpretation purposes (Log-linear transformed: see the Statistical Analysis section for details). The colour of the points in the forest plots reflects the significance of the p-values before and after adjustment for multiple testing using the Benjamini-Hochberg method: empty circles indicate non-significant results (ns), light blue denotes a raw p-value < 0.05, light purple indicates an adjusted p-value < 0.05, and dark purple an adjusted p-value < 0.01. The x-axis incorporates breaks to accommodate wide confidence interval ranges. The colour of the lines represents the antibody isotype or subclass (B). N = 96.
(TIFF)

S6 Fig Association of cytokine concentrations (log10 pg/mL) with antibody levels (log10 MFI). Scatter plots show correlations between antibody and cytokine levels stratified by infection group. Helminth mono-infected individuals are shown in green, and coinfected individuals are shown in orange. Spearman correlation coefficients and p-values are shown in each plot. A linear regression line with a 95% confidence interval is included. The scatter plots continue in S6 Fig (cont).
(ZIP)

S7 Fig Comparison of IgG responses between coinfected and helminth single-infected. Boxplots show helminth-specific IgG responses stratified by infection group. Helminth mono-infected individuals are shown in green, and coinfected individuals are shown in orange. Statistical comparison between groups was performed by the Wilcoxon rank sum test, and the Benjamini-Hochberg method was applied to adjust for multiple comparisons. The plots show both the raw and adjusted p-values.
(JPG)

S8 Fig Comparison of IgG1 responses between coinfected and helminth single-infected. Boxplots show helminth-specific IgG1 responses stratified by infection group. Helminth mono-infected individuals are shown in green, and coinfected individuals are shown in orange. Statistical comparison between groups was performed by the Wilcoxon rank sum test, and the Benjamini-Hochberg method was applied to adjust for multiple comparisons. The plots show both the raw and adjusted p-values.
(JPG)

S9 Fig Comparison of IgG2 responses between coinfected and helminth single-infected. Boxplots show helminth-specific IgG2 responses stratified by infection group. Helminth mono-infected individuals are shown in green, and coinfected individuals are shown in orange. Statistical comparison between groups was performed by the Wilcoxon rank sum test, and the Benjamini-Hochberg method was applied to adjust for multiple comparisons. The plots show both the raw and adjusted p-values.
(JPG)

S10 Fig Comparison of IgG3 responses between coinfected and helminth single-infected. Boxplots show helminth-specific IgG3 responses stratified by infection group. Helminth mono-infected individuals are shown in green, and coinfected individuals are shown in orange. Statistical comparison between groups was performed by the Wilcoxon rank sum test, and the Benjamini-Hochberg method was applied to adjust for multiple comparisons. The plots show both the raw and adjusted p-values.
(JPG)

S11 Fig Comparison of IgG4 responses between coinfected and helminth single-infected. Boxplots show helminth-specific IgG4 responses stratified by infection group. Helminth mono-infected individuals are shown in green, and coinfected individuals are shown in orange. Statistical comparison between groups was performed by the Wilcoxon rank sum test, and the Benjamini-Hochberg method was applied to adjust for multiple comparisons. The plots show both the raw and adjusted p-values.
(JPG)

S12 Fig Comparison of IgA responses between coinfected and helminth single-infected. Boxplots show helminth-specific IgA responses stratified by infection group. Helminth mono-infected individuals are shown in green, and coinfected individuals are shown in orange. Statistical comparison between groups was performed by the Wilcoxon rank sum test, and the Benjamini-Hochberg method was applied to adjust for multiple comparisons. The plots show both the raw and adjusted p-values.
(JPG)

S13 Fig Comparison of IgM responses between coinfected and helminth single-infected. Boxplots show helminth-specific IgM responses stratified by infection group. Helminth mono-infected individuals are shown in green, and coinfected individuals are shown in orange. Statistical comparison between groups was performed by the Wilcoxon rank sum test, and the Benjamini-Hochberg method was applied to adjust for multiple comparisons. The plots show both the raw and adjusted p-values.
(JPG)

S14 Fig Comparison of IgE responses between coinfected and helminth single-infected. Boxplots show helminth-specific IgE responses stratified by infection group. Helminth mono-infected individuals are shown in green, and coinfected individuals are shown in orange. Statistical comparison between groups was performed by the Wilcoxon rank sum test, and the Benjamini-Hochberg method was applied to adjust for multiple comparisons. The plots show both the raw and adjusted p-values.
(JPG)

S15 Fig Comparison of total IgE responses between coinfected and helminth single-infected. Boxplots show total IgE responses stratified by infection group. Helminth mono-infected individuals are shown in green, and coinfected individuals are shown in orange. Statistical comparison between groups was performed by the Wilcoxon rank sum test, and the Benjamini-Hochberg method was applied to adjust for multiple comparisons. The plots show both the raw and adjusted p-values.
(JPG)

S16 Fig Comparison of cytokine responses between coinfected and helminth single-infected. Boxplots show cytokine concentrations stratified by infection group. Helminth mono-infected individuals are shown in green, and coinfected individuals are shown in orange. Statistical comparison between groups was performed by the Wilcoxon rank sum test, and the Benjamini-Hochberg method was applied to adjust for multiple comparisons. The plots show both the raw and adjusted p-values.
(JPG)

S1 Appendix Study database. (CSV)
