# Supplementary material for: Helminth-infected Mozambican children with malaria have increased anaemia, cytokines and helminth-specific antibodies
Source: PLoS Negl Trop Dis. 2026 Jul 13;20(7):e0014485. doi: 10.1371/journal.pntd.0014485 (PMC13362103; doi:10.1371/journal.pntd.0014485)
Supplement: S1 Table — (DOCX) [file pntd.0014485.s001.docx]

**Helminth-infected Mozambican children with malaria have increased anaemia, cytokines and helminth-specific antibodies**

**S1 Table. Helminth antigens**

| **Parasite** | **Antigen** | | **Life stage** | **Rationale** | **Reference** |
| --- | --- | --- | --- | --- | --- |
| **Soil-Transmitted Helminths** |  |  |  |  |  |
| *Necator americanus* | Na-GST-1 | Glutathione S-transferase | Adult stage | Vaccine candidates | [1] |
| *Necator americanus* | Na-APR-1 | Aspartic protease | Adult stage |  | [1] |
| *Necator americanus* | Na-SAA-2 | Surface-associated antigen | Larvae stage |  | [2] |
| *Ancylostoma ceylanicum* | Ay-CP-2 | Cysteine protease | Adult stage |  | [3] |
| *Trichuris muris* | Tm-WAP | Whey acidic protein | Adult stage |  | [4,5] |
| *Trichuris muris* | Tm-16 | Excretory/secretory protein | Adult stage |  | [6] |
| *Ascaris suum* | As-16 | Excretory/secretory protein | Larvae / Adult stage |  | [7,8] |
| *Ascaris suum* | As-37 | Excretory/secretory protein | Larvae / Adult stage |  | [7,9] |
| *Strongyloides stercoralis* | NIE | Recombinant immunodiagnostic antigen | Larvae / Adult stage | Markers of exposure | [10] |
| *Schistosoma mansoni* | Sm-SERPIN | Serine protease inhibitor | Larvae / Adult stage |  | [11] |
| *Schistosoma hematobium* | Sh-SERPIN | Serine protease inhibitor | Larvae / Adult stage |  | [11] |

**References**

1. Zinsou JF, Diemert DJ, Dejon-Agobé JC, Adégbité BR, Honkpehedji YJ, Vodonou KG, et al. Safety and immunogenicity of the co-administered Na-APR-1 and Na-GST-1 hookworm vaccines in school-aged children in Gabon: a randomised, controlled, observer-blind, phase 1, dose-escalation trial. Lancet Infect Dis. 2024 Jul 1;24(7):760–74.

2. Asojo OA, Goud GN, Zhan B, Ordonez K, Sedlacek M, Homma K, et al. Crystallization and preliminary X-ray analysis of Na-SAA-2 from the human hookworm parasite Necator americanus. Acta Crystallogr Sect F Struct Biol Cryst Commun. 2010;66(2):172–6.

3. Wei J, Damania A, Gao X, Liu Z, Mejia R, Mitreva M, et al. The hookworm Ancylostoma ceylanicum intestinal transcriptome provides a platform for selecting drug and vaccine candidates. Parasit Vectors. 2016 Sep 27;9(1).

4. Briggs N, Wei J, Versteeg L, Zhan B, Keegan B, Damania A, et al. Trichuris muris whey acidic protein induces type 2 protective immunity against whipworm. PLoS Pathog. 2018 Aug 1;14(8).

5. Briggs N, Versteeg L, Mejia R, Pollet J, Villar MJ, Zhan B, et al. A Honduran Prevalence Study on Soil-Transmitted Helminths Highlights Serological Antibodies to Tm-WAP49 as a Diagnostic Marker for Exposure to Human Trichuriasis. 2025;

6. Liu C, Chu D, Kalantar-Zadeh K, George J, Young HA, Liu G. Cytokines: From Clinical Significance to Quantification. Vol. 8, Advanced Science. John Wiley and Sons Inc; 2021.

7. de Castro JC, de Almeida L V., Cardoso MS, Oliveira FMS, Nogueira DS, Reis-Cunha JL, et al. Vaccination with chimeric protein induces protection in murine model against ascariasis. Vaccine. 2021 Jan;39(2):394–401.

8. Tsuji N, Miyoshi T, Islam MK, Isobe T, Yoshihara S, Arakawa T, et al. Recombinant Ascaris16‐Kilodalton Protein–Induced Protection against Ascaris suum Larval Migration after Intranasal Vaccination in Pigs. J Infect Dis. 2004 Nov 15;190(10):1812–20.

9. Tsuji N, Kasuga-Aoki H, Isobe T, Arakawa T, Matsumoto Y. Cloning and characterisation of a highly immunoreactive 37 kDa antigen with multi-immunoglobulin domains from the swine roundworm Ascaris suum. Int J Parasitol. 2002 Dec;32(14):1739–46.

10. Arifin N, Hanafiah KM, Ahmad H, Noordin R. Serodiagnosis and early detection of Strongyloides stercoralis infection. Journal of Microbiology, Immunology and Infection. 2019 Jun;52(3):371–8.

11. Tanigawa C, Fujii Y, Miura M, Nzou SM, Mwangi AW, Nagi S, et al. Species-specific serological detection for schistosomiasis by serine protease inhibitor (SERPIN) in multiplex assay. PLoS Negl Trop Dis. 2015;9(8).
