# Supplementary figures and images for: Helminth-infected Mozambican children with malaria have increased anaemia, cytokines and helminth-specific antibodies
Source: PLoS Negl Trop Dis. 2026 Jul 13;20(7):e0014485. doi: 10.1371/journal.pntd.0014485 (PMC13362103; doi:10.1371/journal.pntd.0014485)

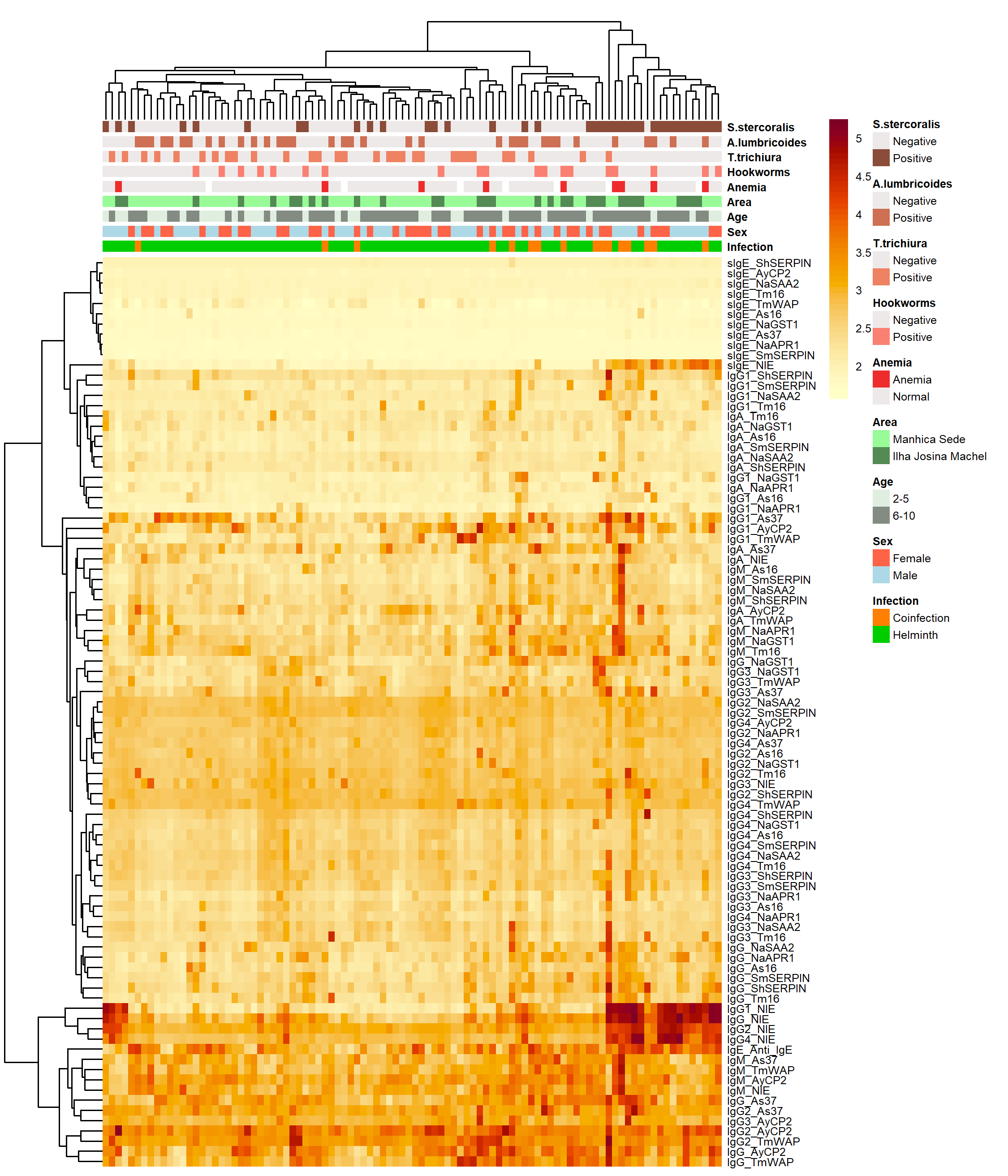

Supplement: S4 Fig — Heatmaps show an overview of antibody (IgG, IgG1-4, IgA, IgM, IgE, and total IgE) responses against helminth antigens. Columns represent study participants, and rows represent antigen-specific antibody levels. Antibody levels (Log10 MFI) are shown on a colour scale from yellow to orange. On top of the heatmap, infection status, sex, age group, area of residence, anaemia, and specific helminth infection status are shown in relation to antibody levels. (TIFF) [file pntd.0014485.s005.tiff]

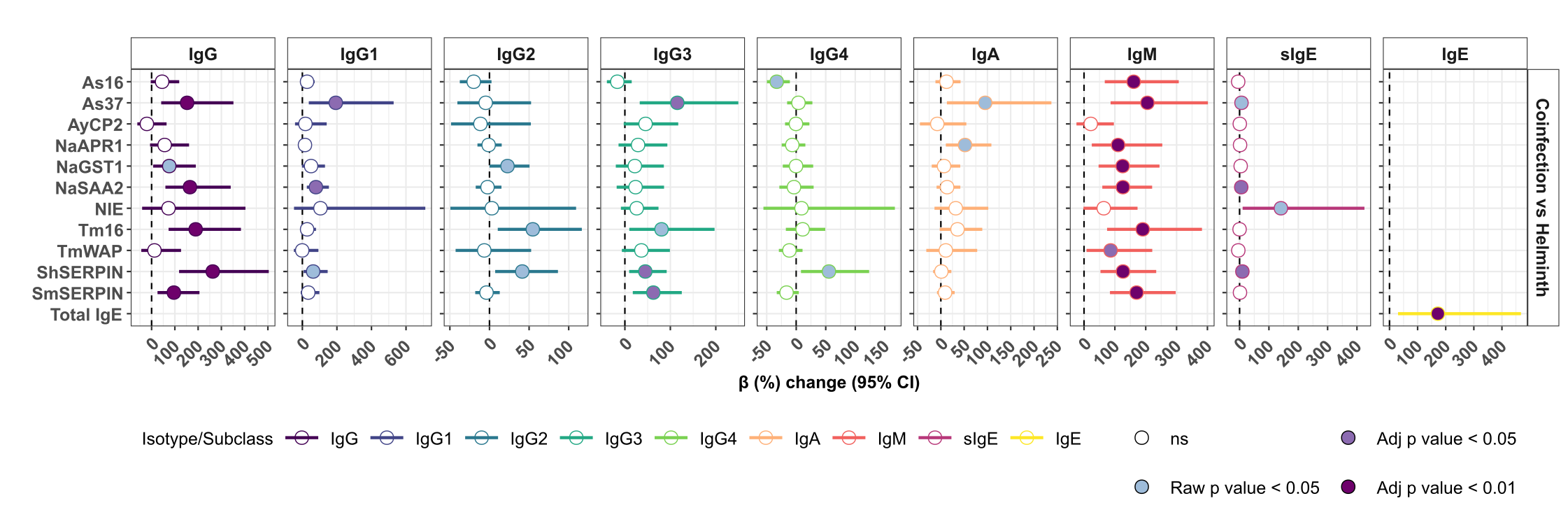

Supplement: S5 Fig — A multivariable linear regression model (adjusted by age, sex, and area) was performed to assess the potential effect of coinfection with malaria and helminths on antibody responses (log10 MFI), considering these possible confounding factors. Forest plots illustrate estimates (points) and 95% confidence intervals (CIs) transformed into percentages for interpretation purposes (Log-linear transformed: see the Statistical Analysis section for details). The colour of the points in the forest plots reflects the significance of the p-values before and after adjustment for multiple testing using the Benjamini-Hochberg method: empty circles indicate non-significant results (ns), light blue denotes a raw p-value < 0.05, light purple indicates an adjusted p-value < 0.05, and dark purple an adjusted p-value < 0.01. The x-axis incorporates breaks to accommodate wide confidence interval ranges. The colour of the lines represents the antibody isotype or subclass (B). N = 96. (TIFF) [file pntd.0014485.s006.tiff]

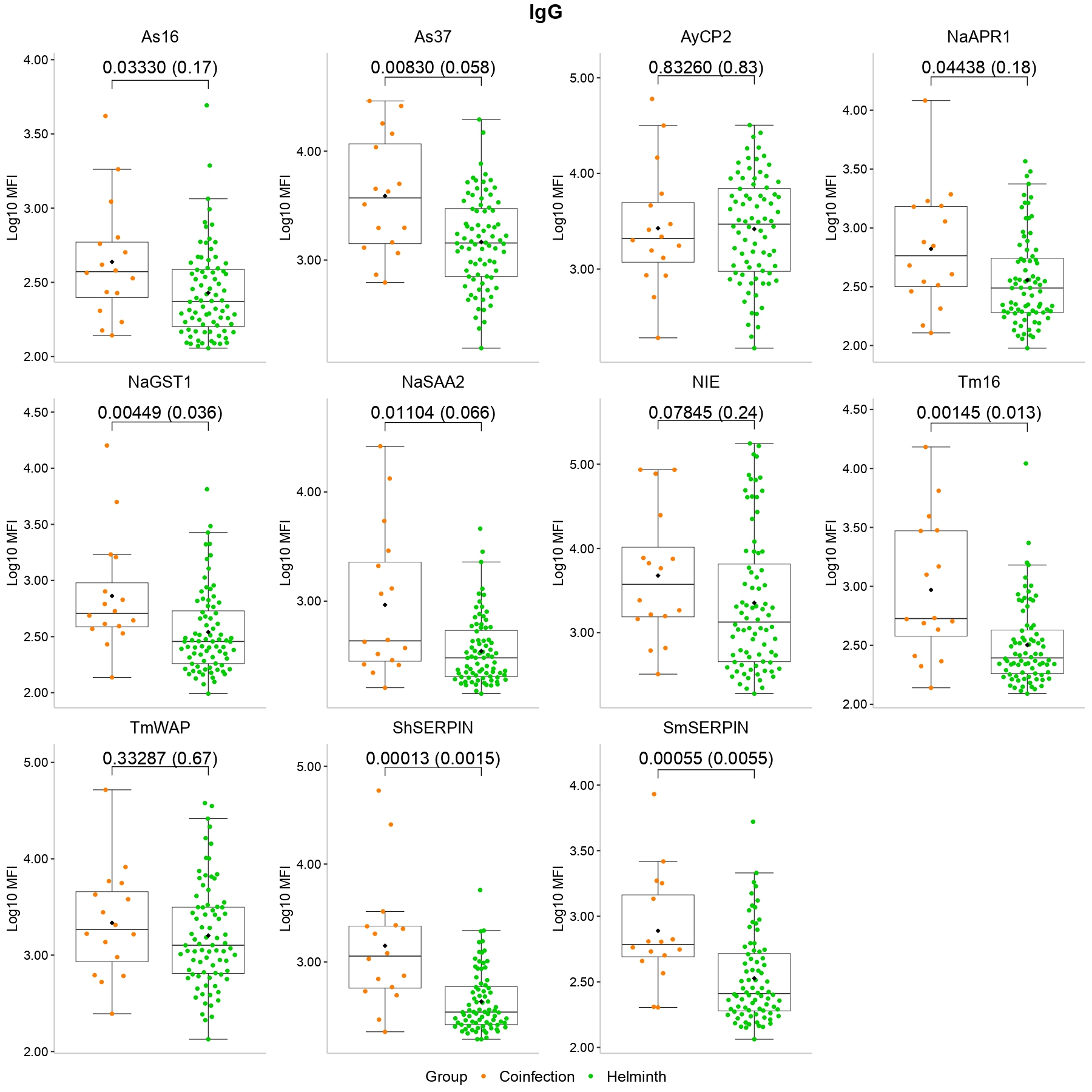

Supplement: S7 Fig — Boxplots show helminth-specific IgG responses stratified by infection group. Helminth mono-infected individuals are shown in green, and coinfected individuals are shown in orange. Statistical comparison between groups was performed by the Wilcoxon rank sum test, and the Benjamini-Hochberg method was applied to adjust for multiple comparisons. The plots show both the raw and adjusted p-values. (JPG) [file pntd.0014485.s008.jpg]

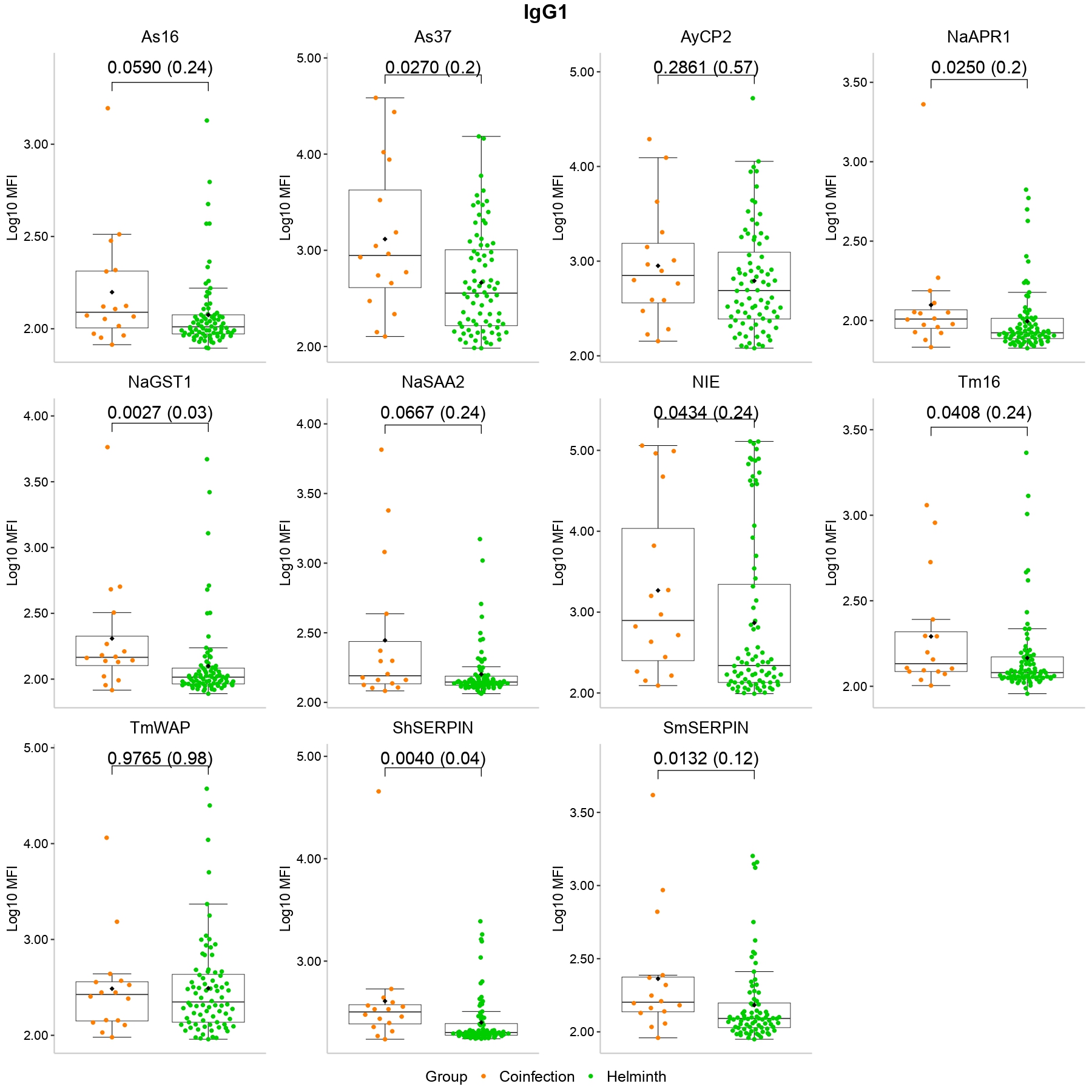

Supplement: S8 Fig — Boxplots show helminth-specific IgG1 responses stratified by infection group. Helminth mono-infected individuals are shown in green, and coinfected individuals are shown in orange. Statistical comparison between groups was performed by the Wilcoxon rank sum test, and the Benjamini-Hochberg method was applied to adjust for multiple comparisons. The plots show both the raw and adjusted p-values. (JPG) [file pntd.0014485.s009.jpg]

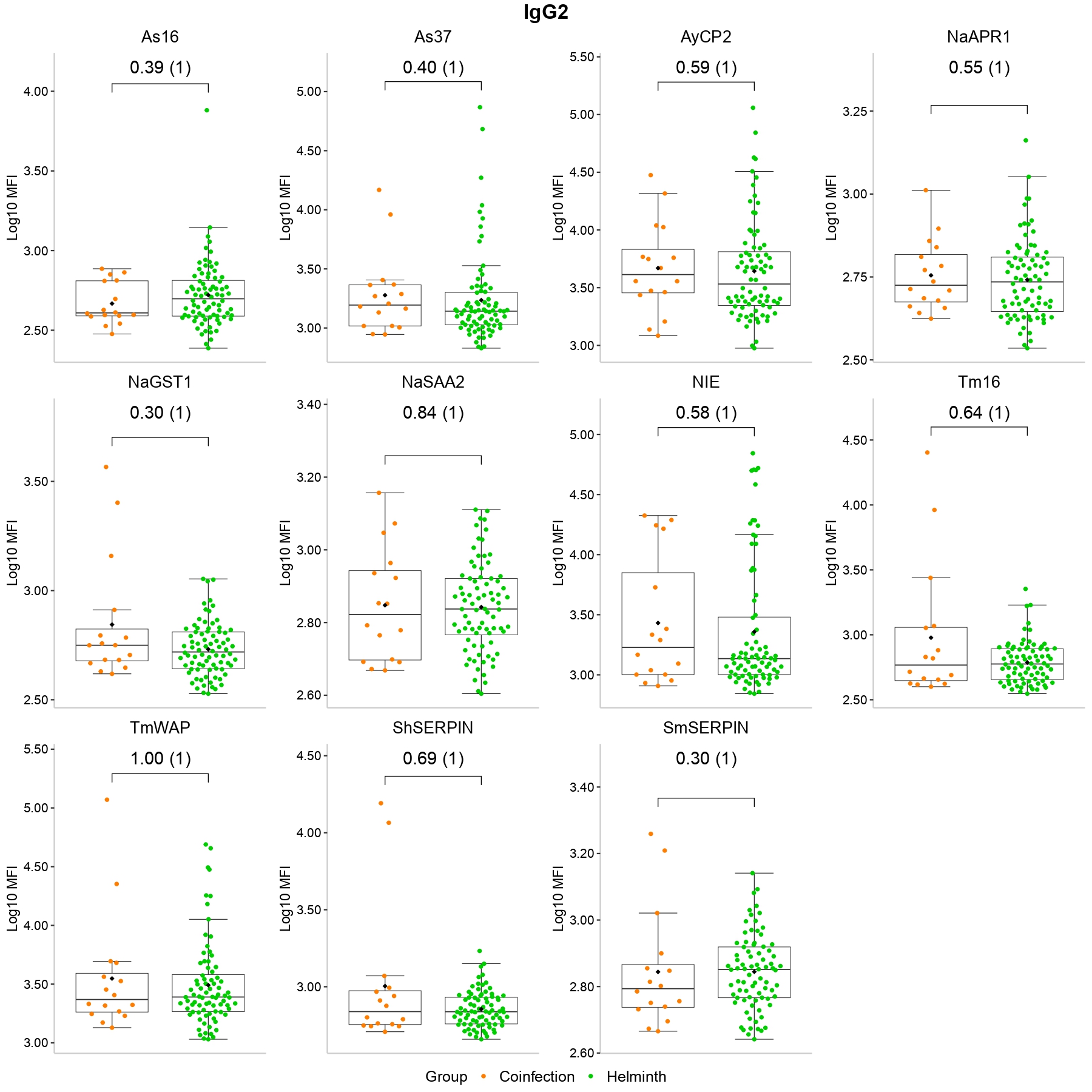

Supplement: S9 Fig — Boxplots show helminth-specific IgG2 responses stratified by infection group. Helminth mono-infected individuals are shown in green, and coinfected individuals are shown in orange. Statistical comparison between groups was performed by the Wilcoxon rank sum test, and the Benjamini-Hochberg method was applied to adjust for multiple comparisons. The plots show both the raw and adjusted p-values. (JPG) [file pntd.0014485.s010.jpg]

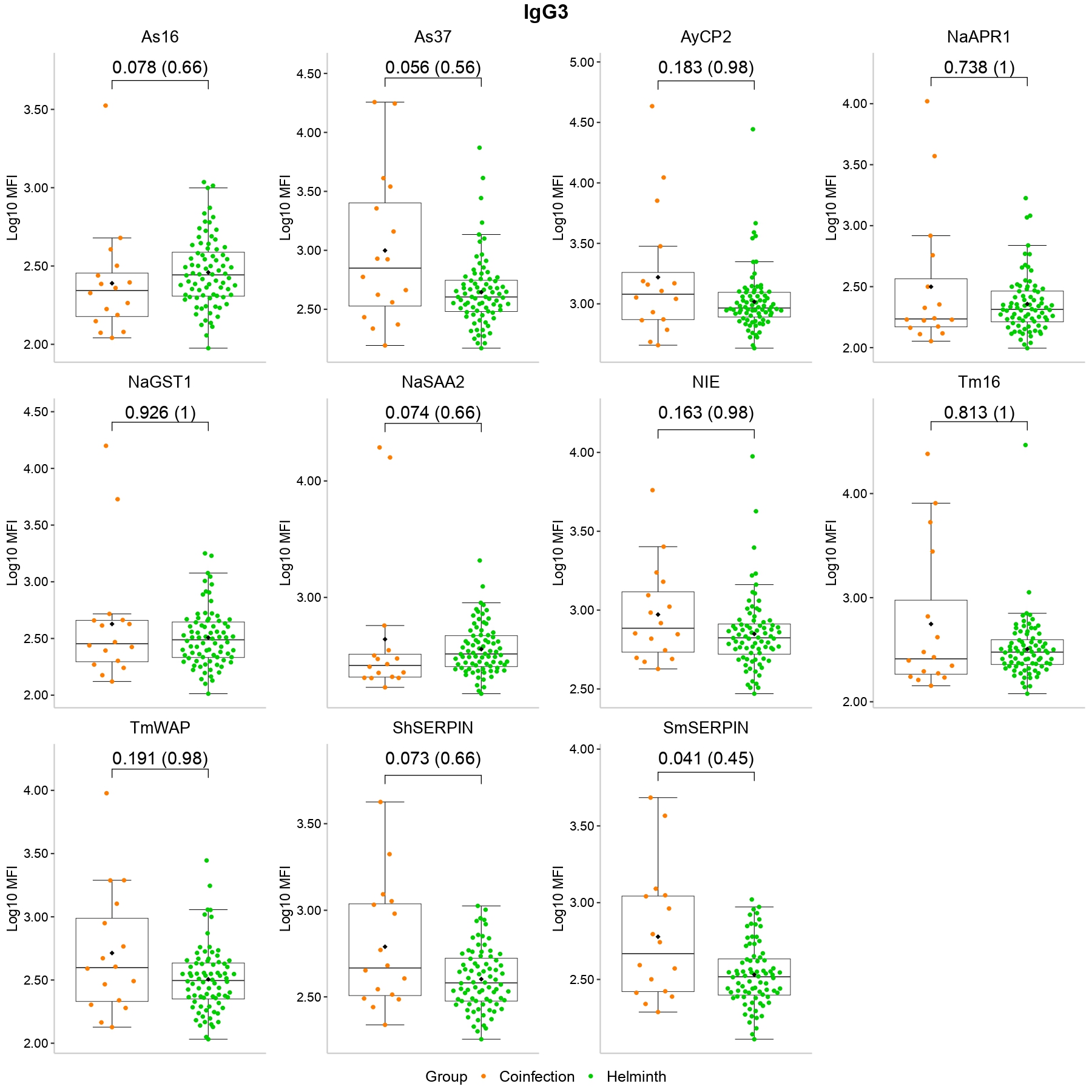

Supplement: S10 Fig — Boxplots show helminth-specific IgG3 responses stratified by infection group. Helminth mono-infected individuals are shown in green, and coinfected individuals are shown in orange. Statistical comparison between groups was performed by the Wilcoxon rank sum test, and the Benjamini-Hochberg method was applied to adjust for multiple comparisons. The plots show both the raw and adjusted p-values. (JPG) [file pntd.0014485.s011.jpg]

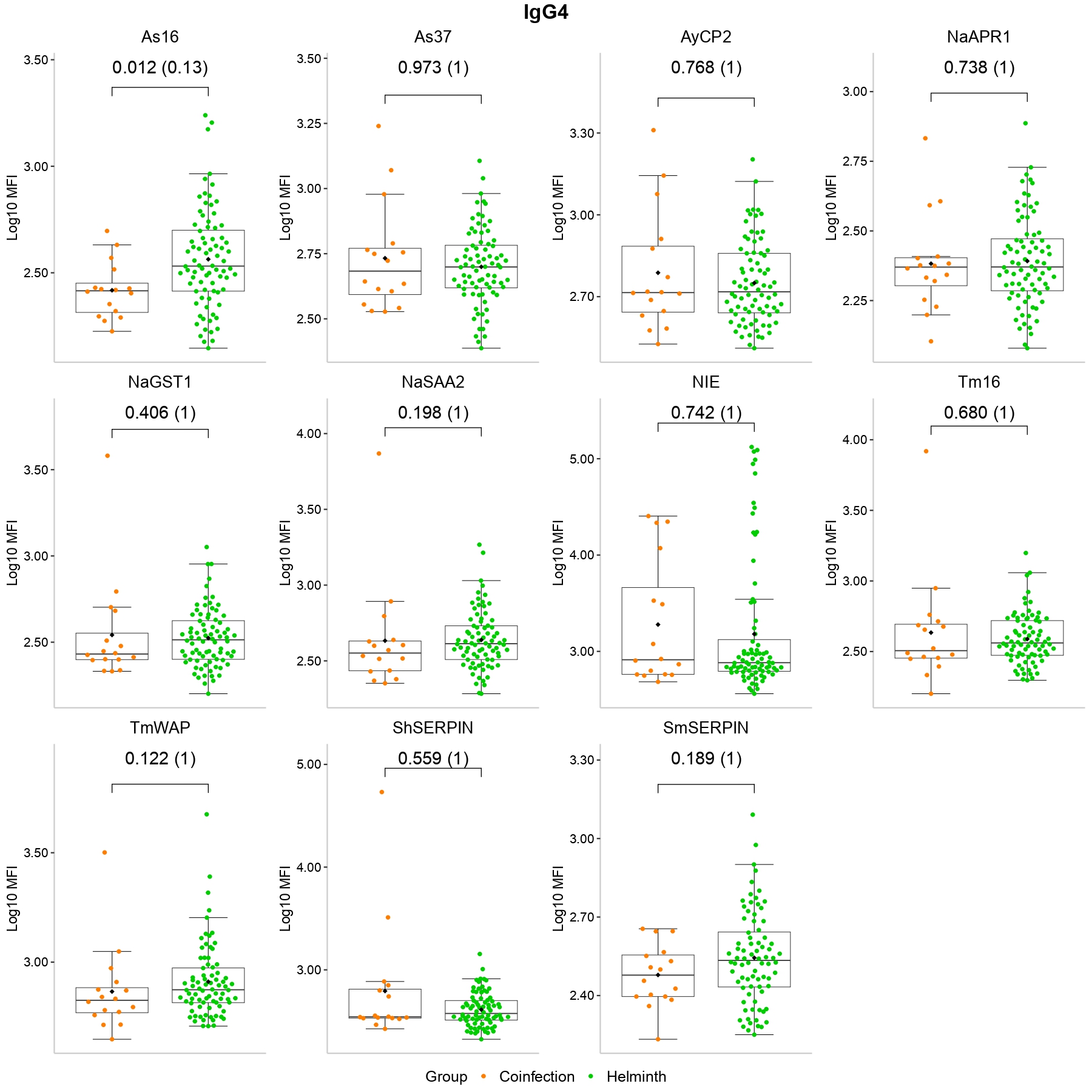

Supplement: S11 Fig — Boxplots show helminth-specific IgG4 responses stratified by infection group. Helminth mono-infected individuals are shown in green, and coinfected individuals are shown in orange. Statistical comparison between groups was performed by the Wilcoxon rank sum test, and the Benjamini-Hochberg method was applied to adjust for multiple comparisons. The plots show both the raw and adjusted p-values. (JPG) [file pntd.0014485.s012.jpg]

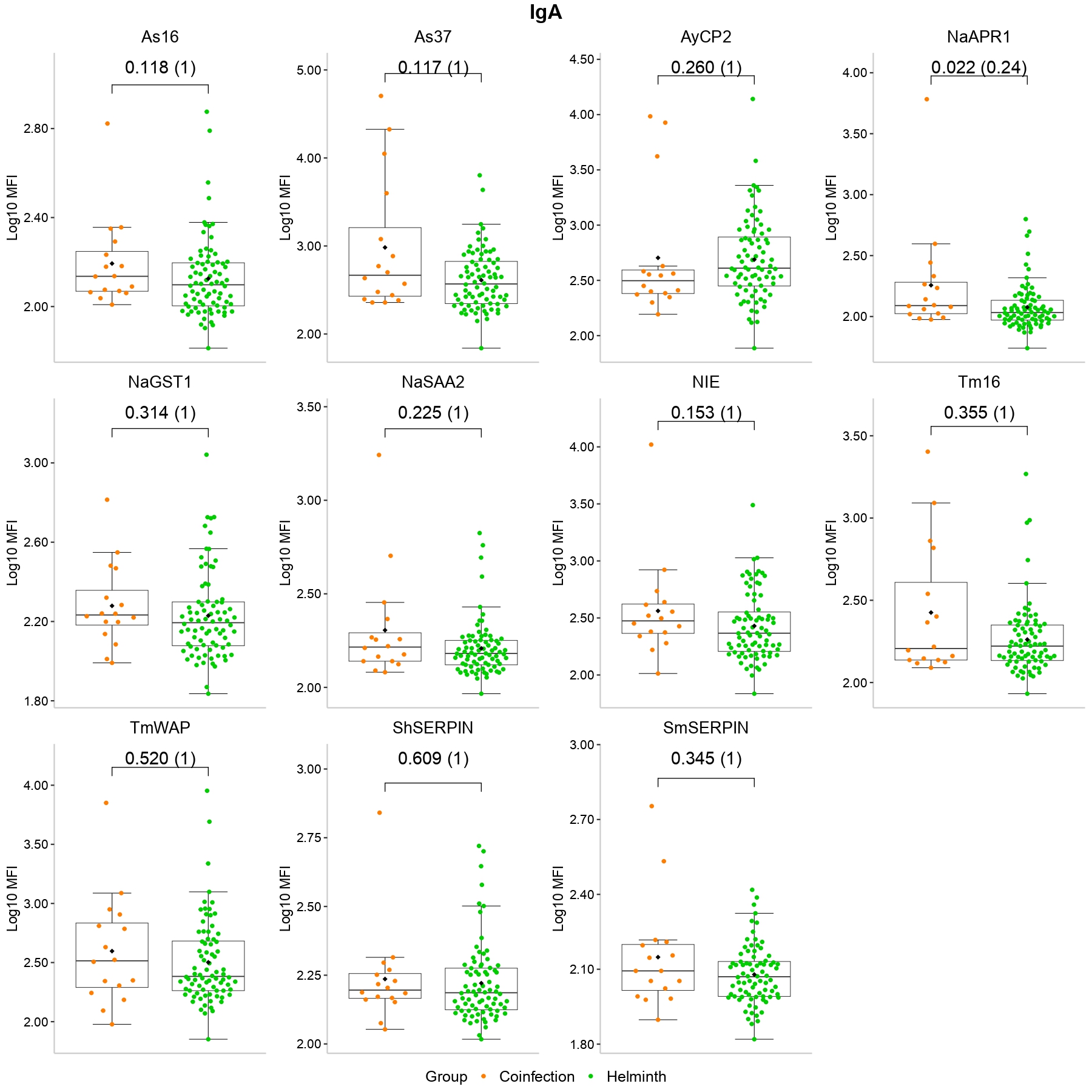

Supplement: S12 Fig — Boxplots show helminth-specific IgA responses stratified by infection group. Helminth mono-infected individuals are shown in green, and coinfected individuals are shown in orange. Statistical comparison between groups was performed by the Wilcoxon rank sum test, and the Benjamini-Hochberg method was applied to adjust for multiple comparisons. The plots show both the raw and adjusted p-values. (JPG) [file pntd.0014485.s013.jpg]

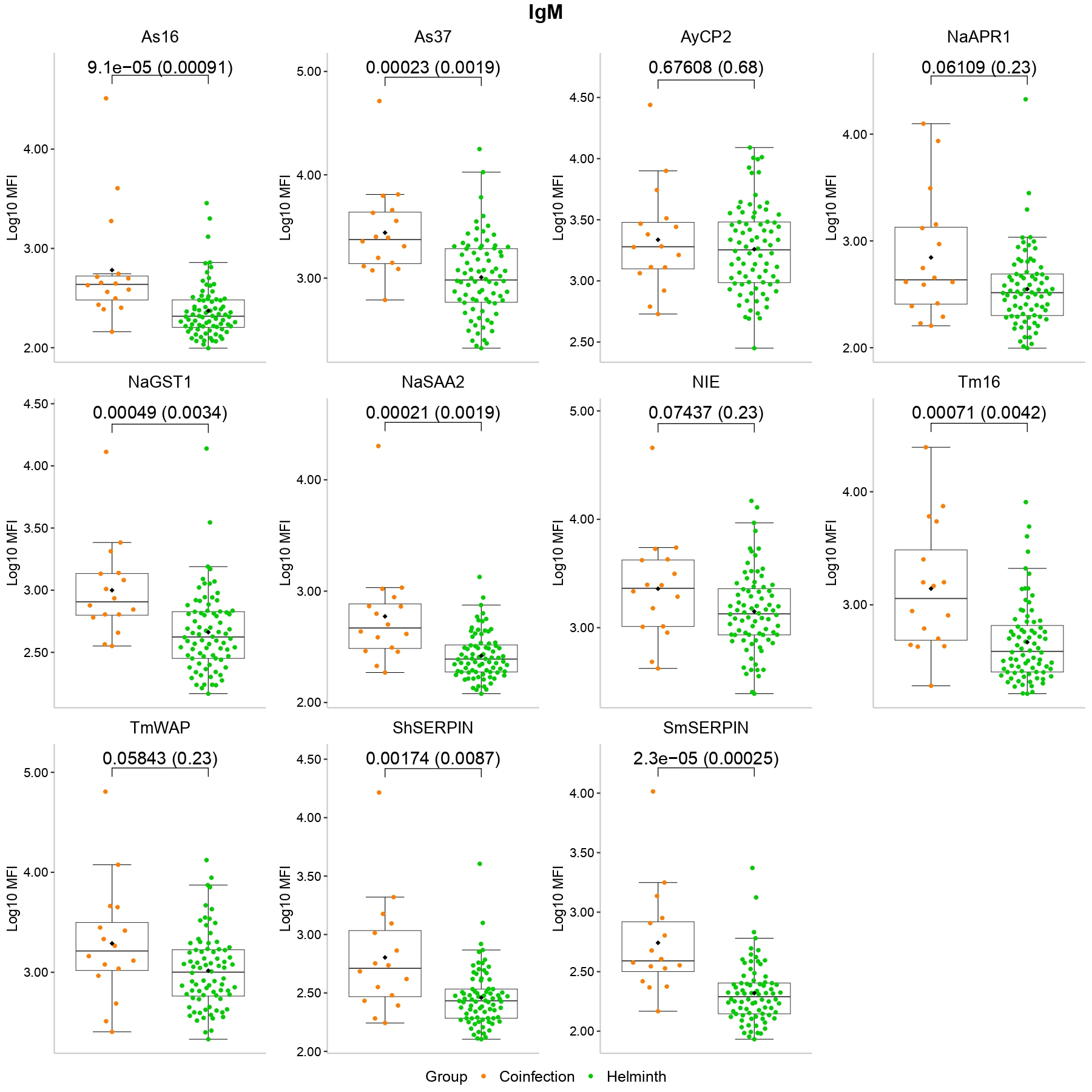

Supplement: S13 Fig — Boxplots show helminth-specific IgM responses stratified by infection group. Helminth mono-infected individuals are shown in green, and coinfected individuals are shown in orange. Statistical comparison between groups was performed by the Wilcoxon rank sum test, and the Benjamini-Hochberg method was applied to adjust for multiple comparisons. The plots show both the raw and adjusted p-values. (JPG) [file pntd.0014485.s014.jpg]

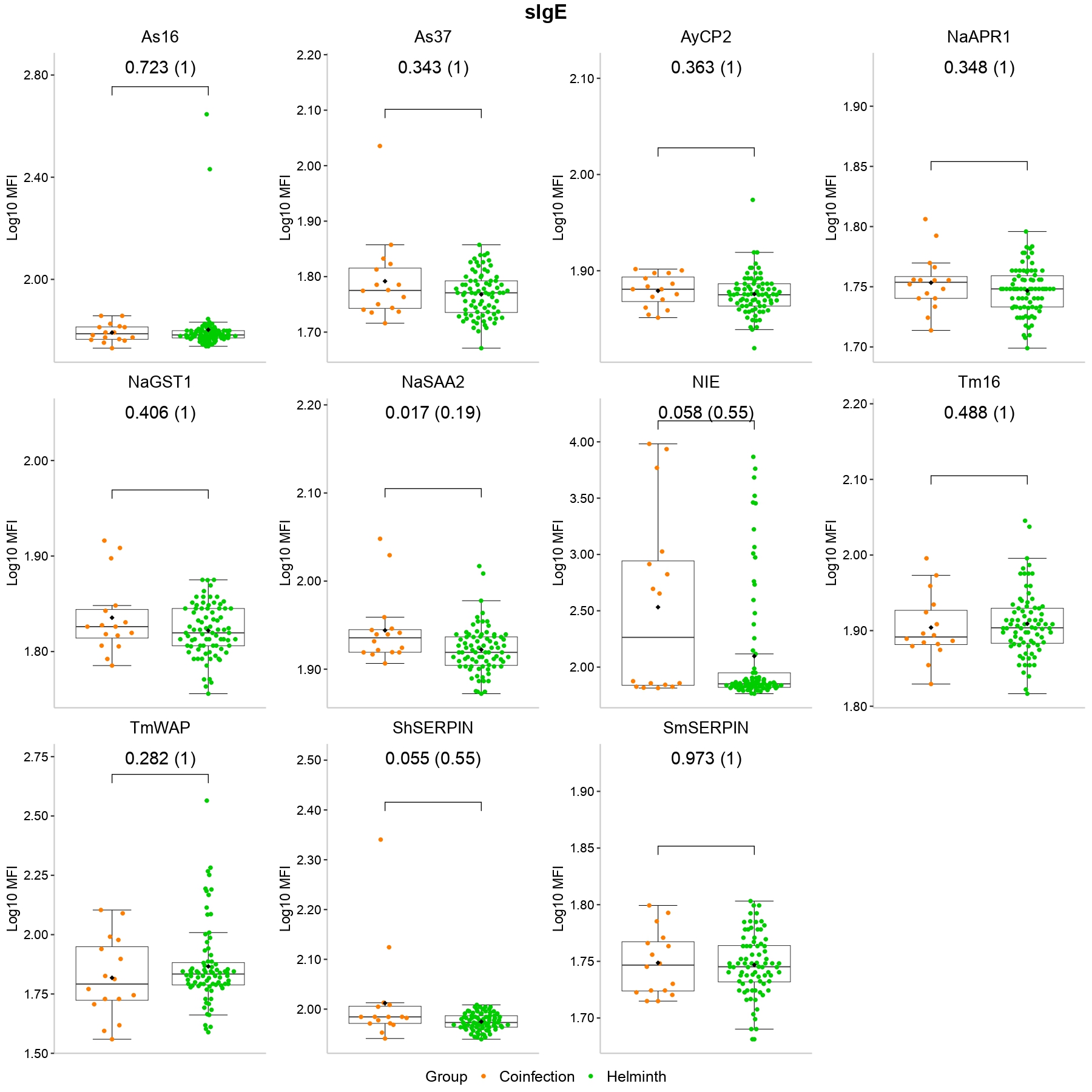

Supplement: S14 Fig — Boxplots show helminth-specific IgE responses stratified by infection group. Helminth mono-infected individuals are shown in green, and coinfected individuals are shown in orange. Statistical comparison between groups was performed by the Wilcoxon rank sum test, and the Benjamini-Hochberg method was applied to adjust for multiple comparisons. The plots show both the raw and adjusted p-values. (JPG) [file pntd.0014485.s015.jpg]

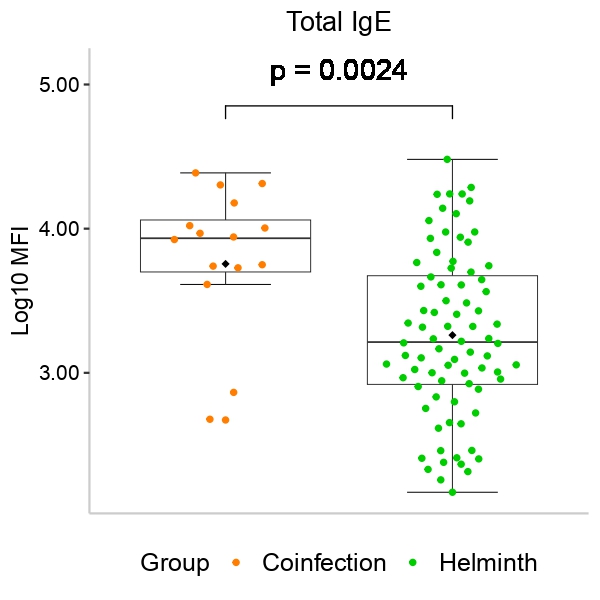

Supplement: S15 Fig — Boxplots show total IgE responses stratified by infection group. Helminth mono-infected individuals are shown in green, and coinfected individuals are shown in orange. Statistical comparison between groups was performed by the Wilcoxon rank sum test, and the Benjamini-Hochberg method was applied to adjust for multiple comparisons. The plots show both the raw and adjusted p-values. (JPG) [file pntd.0014485.s016.jpg]

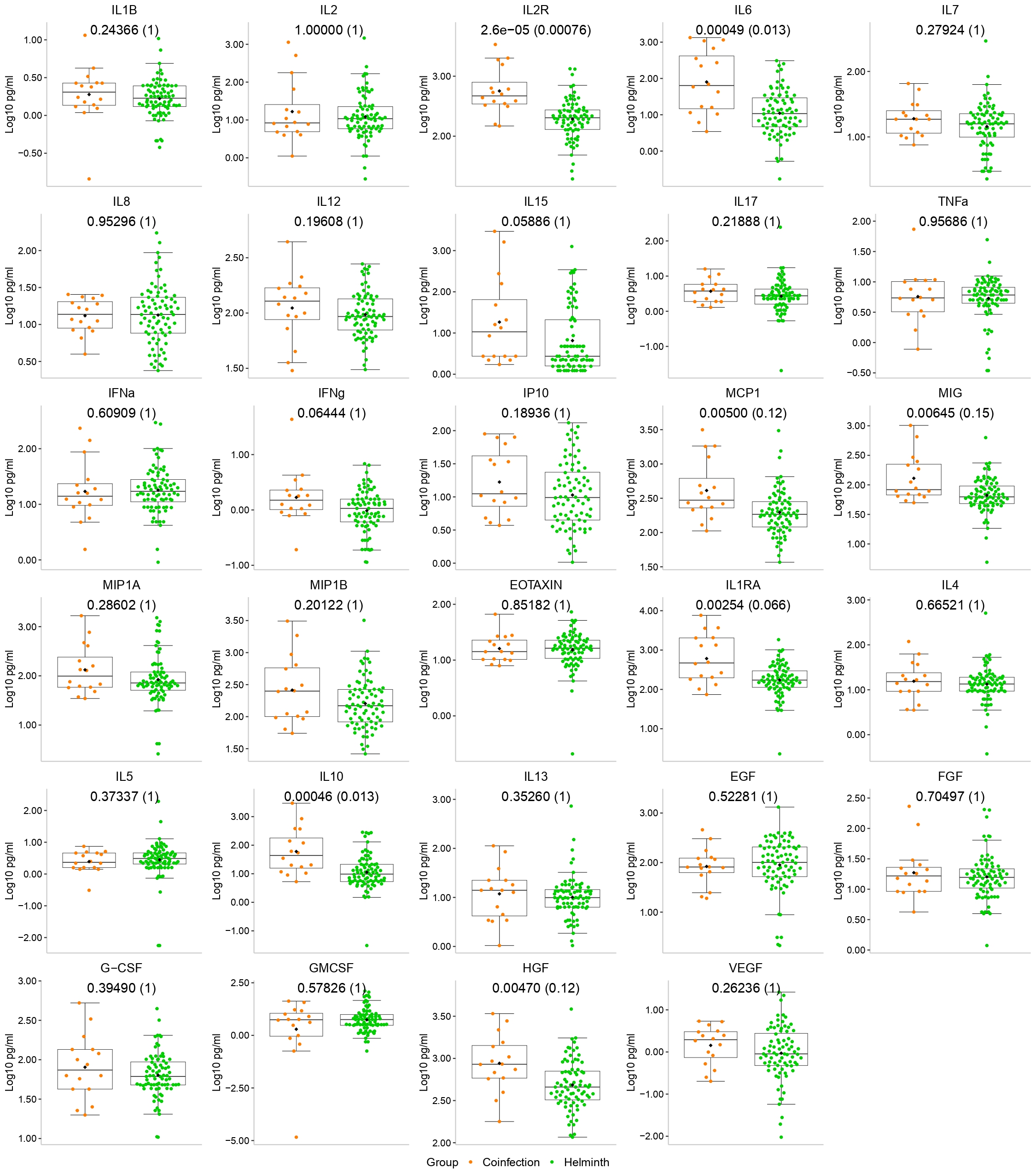

Supplement: S16 Fig — Boxplots show cytokine concentrations stratified by infection group. Helminth mono-infected individuals are shown in green, and coinfected individuals are shown in orange. Statistical comparison between groups was performed by the Wilcoxon rank sum test, and the Benjamini-Hochberg method was applied to adjust for multiple comparisons. The plots show both the raw and adjusted p-values. (JPG) [file pntd.0014485.s017.jpg]
