# Supplementary material for: Helminth-infected Mozambican children with malaria have increased anaemia, cytokines and helminth-specific antibodies
Source: PLoS Negl Trop Dis. 2026 Jul 13;20(7):e0014485. doi: 10.1371/journal.pntd.0014485 (PMC13362103; doi:10.1371/journal.pntd.0014485)
Supplement: Attachment — Submitted filename: Review_Comments.pdf [file pntd.0014485.s019.pdf]

Comments

I would like to thank the authors for this interesting manuscript addressing an important public health topic. The study provides valuable insights into the immunological interactions between malaria and helminth infections in children. However, several revisions would help improve the clarity, methodological transparency, and overall robustness of the manuscript.

Introduction

The manuscript states that over one billion people are affected by NTDs worldwide. Could the authors consider updating it with the most recent WHO Global report on neglected tropical diseases 2025?

The introduction provides a general overview of helminth infections, but the link to malaria co-infection and immune responses remains unclear. The authors may consider strengthening the transition toward the specific focus of the study

You mention that IL-10 levels increase after malaria, but it would be helpful to clarify its dual role: regulating malaria-induced inflammation and modulating the antiparasitic response. Specify that IL-10 limits immunopathology but can promote parasite persistence.

Lignes 97–99: “These interactions that occur in malaria-helminth coinfections…” can be improved. A more fluid formulation would be: “These immunological interactions during malaria helminth coinfections may alter immunological, parasitological and clinical outcomes.”

Lines 110–113: The link with STH vaccines is relevant, but it could be introduced a little better.

Lines 117–123 : you could further clarify the difference between schistosomiasis and STH infections

Study design

The rationale for choosing the hospitals is not explained: is this representative of the general population of children aged 2–10 years in the region?

The recruitment period (August 2015 to December 2016) is long: the seasonal variation of malaria and helminths could influence the results.

Did the authors take seasonality into account in the analysis?No details are given regarding the calculation of the sample size?

Helminth and 151 *P. falciparum* detection

The methods for STH and P. falciparum detection are generally well described and include both microscopy and qPCR, which is a strength. However, several details require clarification.

1- For STH qPCR, the rationale for a Ct cutoff of 38 should be justified, and it is unclear if internal controls were used to assess PCR inhibition.
2- It is also not specified whether each qPCR reaction was performed in duplicate.
3- For P. falciparum, microscopical readings may be subject to inter-observer variability, and qPCR on DBS may have lower sensitivity; controls for extraction and amplification should be described. Clarification on how discordant results between microscopy and qPCR were handled would strengthen reproducibility.

Cytokine, chemokine, and growth factor profiling

The methods for multiplex cytokine measurement are generally well described and include a comprehensive panel of 30 immune mediators. However, several points require clarification.

- While the authors state that the assay volumes were halved without compromising performance, it would strengthen the manuscript to provide validation data confirming this for all analytes
- the handling of values below the limit of detection or above the upper limit of quantification should be described more fully.
- Information on intra-and inter-assay variability, duplicate or triplicate measurements, and batch effect control is missing

Addressing these points would improve reproducibility and interpretation of the immune data.

Statistical analysis

Children who test positive by any method are considered infected. Does this include both microscopy and PCR for STH and malaria? Some mild infections detected only by qPCR may not be biologically equivalent to infections detected by microscopy.

Only two groups are analyzed: helminth-infected vs. coinfected. Why not include a malaria-only group or a non-infected group for reference?

Results

The description of participant groups and STH prevalence is clear and well-organized. However, the coinfected group is very small (n=16, 3.6%), which may limit statistical power for comparisons and regression analyses.

Clarification on whether HIV-positive participants were included in immune analyses, as well as details on the statistical test used for site comparisons and any adjustments for multiple testing, would improve transparency

The description of univariable associations between demographic/clinical variables and plasma cytokines is clear. However, it is unclear whether these associations were adjusted for potential confounders such as infection status, site, sex, or anemia

Discussion

The limitations regarding the small number of coinfected participants, cross-sectional design, and inability to stratify by helminth species are appropriately acknowledged. However, the authors could further emphasize the potential instability of associations due to the small coinfected group and the influence of outliers.
