# Supplementary material for: Helminth-infected Mozambican children with malaria have increased anaemia, cytokines and helminth-specific antibodies
Source: PLoS Negl Trop Dis. 2026 Jul 13;20(7):e0014485. doi: 10.1371/journal.pntd.0014485 (PMC13362103; doi:10.1371/journal.pntd.0014485)
Supplement: Attachment — Submitted filename: Response to Reviewers.pdf [file pntd.0014485.s021.pdf]

Dear Editors,

Thank you for your comments and those of the reviewers. We appreciate the time and effort invested in evaluating our manuscript. We have now carefully revised the manuscript, addressing all the critiques from the editors and reviewers. Below, we provide a detailed point-by-point response to each comment in *italics*, indicating the changes made in the revised manuscript and location. We also took the opportunity to add information that was not sufficiently comprehensive in the original version, like the imputation of low-concentration biomarkers, and the source of antigens for immunoassays, even though reviewers did not raise this, and polished other minor issues.

We hope that the revisions and responses satisfactorily address all concerns, and we thank you for the opportunity to improve our work. We look forward to your prompt further consideration.

With best regards,
Inocencia Cuamba (corresponding author)
On behalf of all authors.

Reviewers' comments:

Reviewer's Responses to Questions

Key Review Criteria Required for Acceptance?

As you describe the new analyses required for acceptance, please consider the following:

Methods

-Are the objectives of the study clearly articulated with a clear testable hypothesis stated?

-Is the study design appropriate to address the stated objectives?

-Is the population clearly described and appropriate for the hypothesis being tested?

-Is the sample size sufficient to ensure adequate power to address the hypothesis being tested?

-Were correct statistical analysis used to support conclusions?

-Are there concerns about ethical or regulatory requirements being met?

Reviewer #1:

no

yes

no

yes

yes

no

Response: *Regarding questions 1 and 3, the objective of this study was to assess the potential impact of coinfection with P. falciparum on helminth-specific immune responses and disease outcomes (lines 154-163). We hypothesised that, due to the opposing Th1 and Th2 immune responses induced by these pathogens, malaria coinfection may likely alter the Th2 response required for helminth expulsion. Additionally, we hypothesised that coinfection could influence clinical outcomes (revised lines 91-103). To address this, we evaluated immune markers and clinical outcomes in coinfected children aged 2 to 10 years attending two hospitals in the Manhiça district, recruited as part of a hospital-based cross-sectional epidemiological study. Among the analysed groups, the number of coinfected participants was relatively small, which we acknowledge as a limitation in the Discussion (lines 590-592).*

Reviewer #2: The study objectives are clearly articulated, with a well-defined and testable hypothesis regarding the impact of malaria coinfection on helminth-specific immunity and clinical outcomes. The cross-sectional, hospital-based design is generally appropriate to explore the stated research questions, although, as acknowledged by the authors, it does not allow for causal inferences. The study population is well described, including age range, recruitment sites, inclusion and exclusion criteria, and relevant clinical characteristics, and is appropriate for addressing the objectives. The overall sample size is reasonable; however, the small number of coinfected participants (n=16) may limit statistical power and increase variability in effect estimates. The statistical analyses, including multivariable regression models and interaction terms, are appropriate for examining cytokine–antibody relationships, with consideration given to multiple comparisons and the exploratory nature of some analyses. Ethical and regulatory requirements were met, with approvals obtained from both the Mozambican National Bioethics Committee and the Spanish Clinical Research Ethics Committee, and written informed consent was obtained from all parents or guardians.

Response: *Thank you for the positive feedback.*

Reviewer #3: The objectives of the study are clearly stated. The design and population is appropriate for the hypothesis tested. The sample size is small (with respect to the 16 co-infected participants), but this is addressed as a limitation in the discussion and clearly stated throughout. The statistical analysis seems robust. Ethical requirements seem appropriate.

A couple of small things:

- In Table 1 there are some missing participants (e.g. only 86-89 participants) for some of the clinical outcomes of fever etc. Please can you provide information in the methods as to why these participants are missing these outcomes, and whether there could be any bias in this data loss. Also – in the figures you say N=96, this is inaccurate in those (e.g. fig 2) when clinical outcomes are used for which you only have N=87.

Response: *Thank you for the positive feedback. Regarding the minor points, not all clinical variables were available for all participants due to incomplete data in the clinical forms, therefore, analyses were performed using available data. This has now been clarified in the Methods section (Lines 300-302). We cannot exclude the possibility of bias due to missing data, and this limitation has now been acknowledged in the Discussion (Lines 585-586). We have also revised the legends of Figures 2, 3, and S2 to accurately reflect the number of participants included in each analysis*.

- Line 186 the chemokine RANTES was excluded due to a substantial number of samples above the ULOQ. Can you quantify "substantial"? Are there any cytokines with a substantial number below the LLOQ, can you provide information on this here?

Response: *For all samples RANTES results clustered close to the ULOQ and was therefore removed from the analyses since it was not informative. For the remaining cytokines, IL-17 showed the highest proportion of samples below the LLOQ (30%). We chose to retain IL-17 because the majority of samples yielded quantifiable values and the analyte displayed consistent patterns in the heatmap analysis. The 30% were imputed by assigning half of the LLOQ (LLOQ/2). We have included this information in the methods section (lines 225-231).*

- Can you fit 200ul in a 374 well plate (line 215)?

Response: *No, the 200 µL/well applies to the 96 well plates while 100 µL/well is the correct quantity of wash buffer used in the 384-well plates. We have included the appropriate information in the methods section (lines 261-262).*

- Were samples randomised between plates?

Response: *Yes, samples were randomised before running the laboratory analysis. We have clarified this in the methods section (lines 255-256).*

<u>Results</u>

-Does the analysis presented match the analysis plan?

-Are the results clearly and completely presented?

-Are the figures (Tables, Images) of sufficient quality for clarity?

Reviewer #1:

yes

yes

no

Response: *We reviewed the tables and figures and ensured they are of enough quality*.

Reviewer #2: The analyses presented are consistent with the methods described in the manuscript and align with the stated study objectives, including the use of multivariable regression, interaction terms, hierarchical clustering, and multivariate approaches. The results are clearly and comprehensively presented, with detailed comparisons between helminth-only and coinfected children that allow readers to follow the key findings. Figures and tables are high quality, clearly labeled, and effectively illustrate the study findings.

Response: *Thank you for your positive feedback*.

Reviewer #3: The results are clearly and completely presented. The figures are high-quality and clear. I am missing any view of the underlying data for the models. Would it be possible to include a supplementary figure(s) with more traditional plots (e.g. boxplot/volcano with individual points per participant) showing helminth vs coinfected? If all outputs is too much, perhaps just including key (significant?) findings.

Response: *Thank you for the positive feedback. We have now provided new boxplots in the supplementary (S7 Fig to S16 Fig).*

- When you state the p value in the results are you always referring to the adjusted p? Could you state this explicitly in the text?

Response: *Yes. Although raw p-values are shown in the plots, the parameters and p-values referred to in the results text are those that remained significant after adjustment for multiple comparisons. This has now been clarified by explicitly stating "adjusted p-value" in the manuscript*.

- In Fig 5 there is a misaligned extra purple square on top of IL8/IgG1

Response: *We have reviewed and corrected the plot*.

<u>**Conclusions**</u>

-Are the conclusions supported by the data presented?

-Are the limitations of analysis clearly described?

-Do the authors discuss how these data can be helpful to advance our understanding of the topic under study?

-Is public health relevance addressed?

Reviewer #1:

yes

no

yes

yes

Response: *We have outlined and expanded the main limitations of the analysis in the Discussion section, including the relatively small number of coinfected participants, which limited statistical power and precluded species-specific stratification, missing data for some clinical variables, and the cross-sectional design, which does not allow for causal inference.*

Reviewer #2: The conclusions drawn in the manuscript are well supported by the data presented, reflecting the observed associations between malaria coinfection, altered cytokine and antibody responses, and clinical outcomes such as anemia. The limitations of the analysis, including the small number of coinfected participants, the cross-sectional design, and the inability to stratify by helminth species, are clearly acknowledged

Response: *Thank you for the positive feedback.*

Reviewer #3: The conclusions are supported by the data presented, and the discussion is good-quality. Limitations and advances of the study are discussed.

Response: *Thank you for the positive feedback.*

<u>Editorial and Data Presentation Modifications?</u>

Use this section for editorial suggestions as well as relatively minor modifications of existing data that would enhance clarity. If the only modifications needed are minor and/or editorial, you may wish to recommend “Minor Revision” or “Accept”.

Reviewer #1: The proposal is well designed scientifically and methodologically. But I have jot down some comments and suggestions

On the title:

There are several studies conducted on similar topic, what is novelty and the values that add the previous study?

Response: *The added value of this study lies in the combined analysis of clinical data, antibody responses, and cytokine responses, in contrast to many previous studies that have examined these components in isolation. For example, several studies report the impact of coinfection on humoral immune responses or inflammatory markers separately without assessing the relationships between these parameters. Furthermore, our study includes all antibody isotypes and subclasses to a broad panel of helminth antigens alongside a wide range of cytokines, enabling the simultaneous analysis of multiple parameters within the same population. Of note, we also measured antigen-specific IgE levels that are relevant in helminth infections but difficult to quantify, and thus not routinely included in most studies. Lastly, much of the existing literature on malaria-helminth coinfections focuses on the effect of helminths on malaria, while the inverse relationship has received less attention. Many studies have also been conducted in animal models, and human data are largely limited to the impact of malaria on Schistosoma infections, with comparatively less information available on its effects on soil-transmitted helminths. We have now included this in the Discussion after limitations (lines 597-604)*.

-In the case of co-infection with malaria and STH infection:

-How do you differentiate whether malaria or STH cause anemia.

Response: *From this study design, we could not determine which pathogen was primarily responsible for the observed anaemia in coinfected children. However, as the prevalence of anaemia was higher in coinfected individuals compared to those with helminth infection alone, we hypothesised that coinfection may have a synergistic effect on the risk of anaemia, likely driven by the malaria infection*.

-How do you measure the severity of the anemia beyond Hgb level or hematocrit level?

Response: *Anaemia severity can be further characterised using additional haematological and biochemical parameters, including red blood cell indices (e.g., MCV, MCH, MCHC), reticulocyte count, and markers of iron status or haemolysis. Clinical assessment and peripheral blood smear findings can also provide insight into the underlying cause and functional severity. However, in this study, we only described anaemia prevalence.*

-I am not comfortable with the naming of the title, it seems like a conclusion to me.

Response: *Our intention was to reflect the study primary findings. We have for now left the title as it was, and defer this point to the editor, as it is usually a matter of journal style to prefer a title that describes what was done vs. a title that summarises the main findings*. *Alternative titles could be:*

Summary of results but less conclusive: "Malaria coinfection is associated with increased anaemia, cytokine levels and helminth-specific antibody responses in helminth-infected Mozambican children".

Descriptive of methods: "Anaemia, cytokine profiles and helminth-specific antibody responses in helminth-infected Mozambican children with and without malaria".

Method and materials

Describe the catchment area of each health facility?

Please describe the current situation of malaria and STHs in the study area.

Response: *To address both of the above comments, we have included a section describing the study area, which reads as follows (lines 173-183):*

Study area

The Manhiça District covers an area of 2,380 km² and has an estimated population of approximately 209,000 inhabitants [31,32]. The district is predominantly rural to semi-rural, with agricultural activities as the main source of income [33]. Malaria transmission is perennial, with peaks occurring during the rainy season from November to April. Malaria remains one of the leading causes of fever in the district, although its incidence has declined over time [34,35]. In Mozambique, helminth infections are widely distributed, with a reported STH prevalence of approximately 13% in Manhiça [36,37]. The district has one main referral facility, the Manhiça District Hospital, which receives patients from peripheral health centres as well as from surrounding areas. The Ilha Josina Machel Health Centre is one such peripheral facility, serving approximately 8,288 inhabitants in the Ilha Josina area [33].

You have not clearly stated your sample size calculation and determination?

Response: *This study was designed as an exploratory investigation within the EcoHeMa project, and used a convenience sampling approach, whereby the sample size was determined by the availability of suitable samples from 441 enrolled participants rather than by a formal a priori calculation.*

How did you allocate your sample size to the two health facilities?

Response: *Participants were recruited based on availability at each site, and all eligible samples from both facilities were included in the analysis.*

Why you did only included children between the age of 2 and 10 for the study?

Response: *The age range of 2–10 years was defined in line with the objectives of the main epidemiological study, which hypothesised that children in this age group are at higher risk of co-exposure due to their behavioural characteristics. We have now clarified the points on sample size and age in the Methods section of the manuscript, which reads as follows (lines 156-161):*

This study was designed as an exploratory investigation within the EcoHeMa project, therefore, the sample size was determined by the number of suitable samples available from enrolled participants. Additionally, the age group was defined to address the objectives of the main epidemiological project, which hypothesised that children aged 2–10 years are potentially the most exposed due to their behavioural characteristics.

How did you control cofounding variables b/c Hgb is subjected to confounding variables?

Response: *We did not perform a formal adjustment for confounding in anaemia analysis, however, we included anaemia and potential confounders, such as age and sex, in the immune response heatmaps and did not observe any relevant clustering within these three variables. Nevertheless, we acknowledge that confounding from unmeasured factors, such as nutritional status, may be present, and we have now included this as a limitation at the end of the Discussion (lines 587-588).*

Give details of statistical considerations in sample size considerations. Also have you checked the effect size?

Response: *As this study was designed as an exploratory analysis within the EcoHeMa project, a formal sample size calculation was not performed, consequently, statistical power and minimum detectable effect sizes were not predefined. We acknowledge the sample size as a limitation in the Discussion section. To address the effect size, we report the regression coefficients (β) with their 95% confidence intervals for all linear regression models, which reflect both the magnitude and direction of each association and serve as a measure of effect size in this context*.

Have you calculated additional percentage for your sample size to take care of drop out?

Response: *As this study was designed as an exploratory analysis within a main project, a formal sample size calculation was not performed, we included all eligible samples available from enrolled participants*.

Why you did not include healthy controls?

Response: *This study was a hospital-based study, therefore, all participants presented with symptoms. We studied current infection and not prior exposure, unlike our prior community-based referred study. As such, children without malaria or helminth infections were not healthy and likely had another condition accounting for their symptoms. We therefore could not use them as healthy controls. We considered that comparing coinfection with single infection was the appropriate comparison for addressing our research questions given the limitations*.

Why didn’t you perform STHs parasite intensity analysis with the severity of anemia?

Response: *Unfortunately, we did not have data on STH parasite intensity, therefore, we were not able to perform analyses assessing the relationship between helminth parasite intensity and anaemia.*

Why didn't you make the study as comparative cross-sectional study?

Response: *We would like to clarify that the ECoHeMa study was indeed designed with predefined comparison groups based on laboratory-confirmed infection status. Participants were prospectively recruited at hospital and assigned to these groups according to diagnostic results, which is consistent with a comparative cross-sectional design. However, as the study was originally conceived as a pilot/exploratory study to assess prevalence and characterise malaria/helminth coinfection patterns, a formal sample size calculation per group was not performed, as previously acknowledged. We acknowledge this as a limitation and have revised the study design description in the Methods section to more accurately reflect its comparative nature (lines 156-161).*

Reviewer #2: (No Response)

Reviewer #3: (No Response)

Summary and General Comments

Use this section to provide overall comments, discuss strengths/weaknesses of the study, novelty, significance, general execution and scholarship. You may also include additional comments for the author, including concerns about dual publication, research ethics, or publication ethics. If requesting major revision, please articulate the new experiments that are needed.

Reviewer #1: None

Reviewer #2: (No Response)

Reviewer #3: This is a technically impressive and well-executed study, with high-quality visualisations and analyses. Whilst the low sample size (n=16 for coinfected) is a clear limitation, it is understandable that large sample sizes may be difficult with the depth of immune outputs analysed. Whilst this study extends and deepens our understanding of responses in co-infected individuals, it does not represent a large 'leap' forward in conceptual understanding. This is discussed further below.

A key area where I think this study is lacking is in not including a malaria-only group. I believe it is well known that acute malaria leads to anaemia and alterations in circulating cytokines. Is it relevant that these individuals have helminth infection, or would these outcomes be exactly the same if comparing acute malaria to no malaria in a helminth uninfected population? Were they outputs (e.g. cytokines, clinical symptoms such as anaemia) run on this group? If so it would greatly improve the novelty of the study to include.

Response: *We thank the reviewer for this pertinent comment. We would like to clarify that the primary research question of the current study was whether malaria coinfection influenced immune responses specific to helminth infection. To address this, the relevant comparison is between helminth-infected children with and without malaria coinfection, which is the analysis we performed. Including a malaria-only group would not directly contribute to answering this specific question and, given the already limited sample size and the large number of immunological variables assessed, would have further complicated the analysis and the interpretation of results*.

We acknowledge, however, that for cytokine profiles it is possible that the effects observed in coinfected children reflect the general impact of acute malaria rather than a specific effect of coinfection. However, the comparison between coinfected individuals and those with malaria alone has been addressed in a separate paper, and we did see differences in the coinfected vs. the malaria only group in terms of cytokines and clinical outcomes.*

**Cuamba I, Santano R, Grau-Pujol B, Vidal M, Aguilar R, Cossa A, Jairoce C, Mejia R, Nhabomba A, Muñoz J, Moncunill G, Dobaño C. Helminth coinfections mitigate clinical, parasitological, and immune outcomes in Mozambican children with malaria. BMC Med. 2026 Mar 31;24(1):189. doi: 10.1186/s12916-026-04827-7. PMID: 41918093; PMCID: PMC13037013.*

Note – the above criticism is less applicable to helminth-specific antibody outputs (although still interesting – presumably these children could have helminth-specific antibodies from a prior helminth infection, does malaria increase those? This may fit your polyclonal b cell activation hypothesis (line 517).)

Response: *It is plausible that these children may have helminth-specific antibodies from prior helminth infections, and that malaria coinfection could lead to broad B cell stimulation, including activation of memory B cells specific to helminths, resulting in increased antibody levels. However, exploring this question would require additional stratification, which is limited by the current sample size and would reduce statistical power. Moreover, in the absence of longitudinal data, it is difficult to determine the timing of previous helminth infections, making such analyses uncertain and beyond the scope of the present study.*

The authors have previously shown that malaria co-infection increases helminth-specific antibodies in coinfected individuals (ref 50). I am surprised this reference appears so late in the text, and should be highlighted in the introduction. The authors should be more explicit as to how the current work extends the findings of ref 50 (and other relevant literature?)– e.g. isotypes? Study population? antigens tested?

Response: *The reviewer is right and we thank you for pointing this out. We have now referenced our previous work in the Introduction (lines 141-143), as suggested, and have included an explanation of the added value in the Discussion, which reads as follows (lines 597-604):*

Specifically, the added value of this study lies in the combined analysis of clinical data, antibody (including antigen-specific IgE) and cytokine responses, whereas many previous studies have examined these components in isolation. Furthermore, our study includes a broad panel of helminth antigens alongside a wide range of cytokines, enabling the simultaneous analysis of multiple parameters within the same population. Lastly, while much of the existing literature on malaria-helminth coinfections has focused on the effect of helminths on malaria, the inverse relationship has received comparatively less attention.

PLOS authors have the option to publish the peer review history of their article (what does this mean?). If published, this will include your full peer review and any attached files.

If you choose “no”, your identity will remain anonymous but your review may still be made public.

Do you want your identity to be public for this peer review? For information about this choice, including consent withdrawal, please see our Privacy Policy.

Reviewer #1: No

Reviewer #2: No

Reviewer #3: No

Comments from the attached PDF File

Comments

I would like to thank the authors for this interesting manuscript addressing an important public health topic. The study provides valuable insights into the immunological interactions between malaria and helminth infections in children. However, several revisions would help improve the clarity, methodological transparency, and overall robustness of the manuscript.

<u>Introduction</u>

The manuscript states that over one billion people are affected by NTDs worldwide. Could the authors consider updating it with the most recent WHO Global report on neglected tropical diseases 2025?

Response: *Thank you for the positive feedback. We have updated the manuscript to reflect the most recent WHO Global Report on Neglected Tropical Diseases (2025)*.

The introduction provides a general overview of helminth infections, but the link to malaria co-infection and immune responses remains unclear. The authors may consider strengthening the transition toward the specific focus of the study.

Response: *In lines 100-117, we highlight that, in murine models, malaria coinfection has been shown to potentially suppress Th2 immune responses, which are required for helminth expulsion and may therefore influence helminth burden and associated morbidity. Furthermore, we note that it is hypothesised that an effective helminth vaccine would need to induce strong Th2 responses, therefore, malaria coinfection, which is typically associated with a strong Th1 response, may also have a suppressive effect on vaccine-induced immunity. We have revised the text to strengthen the transition in the introduction's last paragraph*.

You mention that IL-10 levels increase after malaria, but it would be helpful to clarify its dual role: regulating malaria-induced inflammation and modulating the antiparasitic response. Specify that IL-10 limits immunopathology but can promote parasite persistence.

Response: *We have clarified the dual role of IL-10 in line 96.*

Lignes 97–99: “These interactions that occur in malaria-helminth coinfections…” can be improved. A more fluid formulation would be: “These immunological interactions during malaria helminth coinfections may alter immunological, parasitological and clinical outcomes.”

Response: *We have included the suggested correction in lines 98-99, in the framework of the study hypothesis that is now made more explicit*.

Lines 110–113: The link with STH vaccines is relevant, but it could be introduced a little better.

Response: *We have now edited this section, which now reads as follows from lines 111 to 117: Furthermore, there has been increasing interest in developing helminth vaccines as an additional control measure in the push toward helminth elimination. This has thus far proven challenging due to the complexity of these pathogens and host-parasite interactions [23]. It is thought that an ideal STH vaccine should elicit a robust Th2 immune response [24]. Given that these vaccines are being developed for use in countries endemic to several Th1-inducing infections, including malaria, it is essential to determine whether coinfection could also suppress vaccine responses to worm antigens*.

Lines 117–123 : you could further clarify the difference between schistosomiasis and STH infections.

Response: *We have revised this section, which now reads as follows (lines 128-132):*

Both STH infections and schistosomiasis are characterised by predominantly Th2-biased and regulatory immune responses during chronic infection, however, schistosomiasis typically exhibits a more pronounced biphasic response, with an early Th1 phase followed by a strong Th2 shift driven by egg deposition [27,28]. Therefore, the need for species-specific studies as the outcomes may likely be distinct.

Study design

The rationale for choosing the hospitals is not explained: is this representative of the general population of children aged 2–10 years in the region?

Response: *The Manhiça District has one main referral facility, Manhiça District Hospital, which receives patients from peripheral health centres as well as surrounding areas, allowing the capture of patients from across the district. In addition, the Manhiça Health Research Centre is located in close proximity to the district hospital, making hospital-based recruitment more logistically feasible, particularly when samples need to be collected and processed within a limited timeframe. However, studies may also extend to peripheral health centres. In this study, Ilha Josina Machel Health Centre, one of the peripheral facilities, was included because Ilha Josina is known to be a malaria hotspot in the region and presents conditions associated with an increased risk of helminth infections. Due to these differences, analyses were adjusted by study area where applicable. The sample may not be fully representative of the general population of children aged 2–10 years in the region, rather, it reflects children seeking care at these health facilities. We have added a description of the study area in the manuscript.*

The recruitment period (August 2015 to December 2016) is long: the seasonal variation of malaria and helminths could influence the results.

Did the authors take seasonality into account in the analysis? No details are given regarding the calculation of the sample size?

Response*: We did not account for seasonal variation in our analyses, however, we acknowledge that malaria transmission may vary seasonally, particularly with peaks during the rainy season, and that this may influence infection prevalence and immune responses. We have now included this as a limitation in the manuscript Discussion (lines 588-590).*

Regarding the sample size, a formal calculation was not performed, instead, we included all eligible samples available from enrolled participants, as this study was designed as an exploratory analysis

within a main project. We have now addressed this in the study design section of the manuscript (lines 156-161).

Helminth and *P. falciparum* detection

The methods for STH and *P. falciparum* detection are generally well described and include both microscopy and qPCR, which is a strength. However, several details require clarification.

For STH qPCR, the rationale for a Ct cutoff of 38 should be justified, and it is unclear if internal controls were used to assess PCR inhibition.

Response: *This cutoff was established based on the detection limits of each assay, determined using serial dilutions of parasite-specific plasmid controls. The Ct threshold of 38 corresponded to the lower limit of the standard curves, beyond which additional plasmid dilutions were no longer detectable. We have now justified this in the Methods section (lines 194-197).*

It is also not specified whether each qPCR reaction was performed in duplicate.

Response: *The qPCR reactions were performed in duplicate. This has now been clarified in the manuscript's Methods section (line 189).*

For *P. falciparum*, microscopical readings may be subject to inter-observer variability, and qPCR on DBS may have lower sensitivity; controls for extraction and amplification should be described. Clarification on how discordant results between microscopy and qPCR were handled would strengthen reproducibility.

Responses: *We agree that microscopic readings may be subject to inter-observer variability, and this was accounted for in the laboratory. According to internal procedure, microscopy readings are performed independently by two trained microscopists, and a third reading is conducted in cases of discordance to resolve discrepancies. We added these details to the manuscript in lines 199-201.*

Regarding controls, blanks and negative controls were used to control for extraction and amplification. We have now specified this in the manuscript (lines 203-204).

Regarding discordant results between microscopy and qPCR, the latter was performed only on samples that were negative by microscopy.
