# Supplementary material for: Helminth-infected Mozambican children with malaria have increased anaemia, cytokines and helminth-specific antibodies
Source: PLoS Negl Trop Dis. 2026 Jul 13;20(7):e0014485. doi: 10.1371/journal.pntd.0014485 (PMC13362103; doi:10.1371/journal.pntd.0014485)
Supplement: Attachment — Submitted filename: Response_to_Reviewers_auresp_2.pdf [file pntd.0014485.s022.pdf]

Dear Editors,

Thank you for your second round of comments and those of the reviewers. We appreciate again the time and effort invested in evaluating our manuscript. We have carefully revised the manuscript and addressed all new comments raised by the editors and reviewers. Below, we provide a detailed point-by-point response to each comment in *italics*, indicating the corresponding changes made in the revised manuscript and their location. Some comments did not lead to further changes to the manuscript, as they had already been addressed in the previous round of review.

We hope that the revisions and responses satisfactorily address all concerns, and we thank you for the opportunity to improve our work. We look forward to your prompt further consideration.

With best regards,

Inocência Cuamba (corresponding author)

On behalf of all authors.

Reviewers' comments:

Reviewer's Responses to Questions

Key Review Criteria Required for Acceptance?

As you describe the new analyses required for acceptance, please consider the following:

Methods

-Are the objectives of the study clearly articulated with a clear testable hypothesis stated?

-Is the study design appropriate to address the stated objectives?

-Is the population clearly described and appropriate for the hypothesis being tested?

-Is the sample size sufficient to ensure adequate power to address the hypothesis being tested?

-Were correct statistical analysis used to support conclusions?

-Are there concerns about ethical or regulatory requirements being met?

Reviewer #2: The study addresses an important question on malaria–helminth coinfection and immune responses in children. The objectives are relevant, but a clear testable hypothesis is lacking.

We thank the reviewer for the positive comments. In response to this comment, we have revised the Introduction to explicitly include a testable hypothesis, thereby clarifying the expected outcomes (Lines 140-146).

"Given the potential impact of malaria coinfection on helminth immune responses, we hypothesised that the Th1-dominant immune environment induced by Plasmodium infection would suppress Th2-associated anti-helminth responses in children, including helminth-specific IgE and IgG4 antibody levels and type 2 cytokine concentrations, while elevating pro-inflammatory cytokines. Additionally, we hypothesised that these immunological alterations would be accompanied by worse clinical outcomes than in children with helminth infection alone."

The cross-sectional design is appropriate for associations but does not allow causal inference.
We acknowledge that the cross-sectional design does not permit causal inference. This limitation has been recognised in the Discussion section (Lines 601–602).

The hospital-based recruitment may introduce selection bias.
We agree and have now acknowledged the limitations of hospital-based recruitment and the potential for selection bias in the Discussion (Lines 586-592).

A major limitation is the very small number of coinfected participants (n = 16), raising concerns about statistical power and robustness of the analyses.
We have acknowledged the small number of coinfected participants as a limitation in the Discussion section (Lines 598-599), which limited statistical power and some analyses. In addition, we would like to clarify that this was an exploratory study; therefore, we proceeded with the analyses despite the small number of coinfected participants. We hope that the findings from this work will be informative for the design of future, larger studies in which these observations can be further evaluated and validated.

Although appropriate statistical methods were used, further clarification is needed regarding adjustment for key confounders. Overall, the study is of interest but methodological and statistical limitations should be addressed before firm conclusions can be drawn.
Thanks for considering the study of interest. To account for potential confounding in the analyses assessing the effect of P. falciparum coinfection on immune responses, multivariable linear regression models were adjusted for age, sex, and area of residence based on prior knowledge and directed acyclic graph (DAG) informed confounder assessment (Line 330-332). Furthermore, we acknowledged in the Discussion that residual confounding from unmeasured variables may remain.

Reviewer #3: (No Response)

Results

-Does the analysis presented match the analysis plan?

-Are the results clearly and completely presented?

-Are the figures (Tables, Images) of sufficient quality for clarity?

Reviewer #2: The analyses generally appear consistent with the statistical methods described. The results are clearly presented overall, although a clearer distinction between findings that remain significant after multiple-testing correction and exploratory findings would improve interpretation.

The parameters and p-values referred to in the results text are those that remained significant after adjustment for multiple comparisons. This had also been mentioned in the previous set of comments, so we edited the manuscript and clarified by explicitly stating "adjusted p-value" in the manuscript.

A major concern remains the small number of coinfected participants, which may affect the robustness of some analyses.

We have acknowledged the small number of coinfected participants as a limitation in the Discussion section (Lines 598-599), which limited statistical power and some analyses.

In addition, the quality of several figures is suboptimal. The image resolution should be improved, as some text, labels, and graphical elements are difficult to read. Higher-resolution figures are needed to ensure clarity and proper evaluation of the results.

The figures were prepared in accordance with the PLOS Neglected Tropical Diseases guidelines and were checked using the journal's NAAS tool to ensure compliance with the requirements. Nevertheless, we have carefully re-examined all figures to ensure that resolution, labels, and graphical elements are clear and of good quality.

Reviewer #3: (No Response)

Conclusions

-Are the conclusions supported by the data presented?

-Are the limitations of analysis clearly described?

-Do the authors discuss how these data can be helpful to advance our understanding of the topic under study?

-Is public health relevance addressed?

Reviewer #2: The conclusions are generally supported by the data but are somewhat stronger than warranted given the cross-sectional design and the small number of coinfected participants. The limitations are acknowledged, but key issues such as potential confounding and limited statistical power could be more fully emphasized. The authors discuss the relevance of the findings for understanding malaria–helminth interactions, and the public health implications are mentioned, although they remain largely speculative and should be toned down.

We acknowledge that the cross-sectional design, the small number of coinfected participants, and the potential for residual confounding limit the strength of causal inference and the statistical power of the analyses, and we have ensured that these limitations are included in the Discussion.

We have revised the manuscript to guarantee the use of moderate language in the Discussion and Conclusions to avoid overstatement.

Reviewer #3: (No Response)

Editorial and Data Presentation Modifications?

Use this section for editorial suggestions as well as relatively minor modifications of existing data that would enhance clarity. If the only modifications needed are minor and/or editorial, you may wish to recommend “Minor Revision” or “Accept”.

Reviewer #2: (No Response)

Reviewer #3: (No Response)

Summary and General Comments

Use this section to provide overall comments, discuss strengths/weaknesses of the study, novelty, significance, general execution and scholarship. You may also include additional comments for the author, including concerns about dual publication, research ethics, or publication ethics. If requesting major revision, please articulate the new experiments that are needed.

Reviewer #2: The topic is relevant and original. The study addresses a relatively underexplored question: the impact of malaria co-infection on anti-helminth immune responses in children. The immunological approach is ambitious, and the results appear to be interesting. However, several points require clarification.

We thank the reviewer for the positive assessment.

Introduction

1) The authors should moderate this statement and more clearly define the specific gap addressed by the present study (e.g., effects on antibody responses against vaccine candidate antigens and multiplex immune profiling in children).

We are not entirely certain which specific statement the reviewer is referring to. However, the knowledge gap we intended to address was the potential impact of malaria coinfection on helminth-specific immune responses and clinical outcomes, an area that has received considerably less attention than the impact of helminth coinfection on immune responses to other pathogens (Lines 127–131). To address this gap, we analysed helminth-specific antibody responses using a panel of antigens that included several helminth antigens, including some vaccine candidate antigens, together with a multiplex panel of immune markers that could provide insights into Th1, Th2, and other immune response profiles. We have revised the Introduction to more clearly articulate the testable hypotheses which relates to the knowledge gap addressed.

2) The introduction would benefit from a more balanced discussion of the potential mechanisms underlying both suppressive and enhancing effects of malaria coinfection

We agree that both suppressive and enhancing effects of malaria coinfection on helminth-specific immune responses merit a balanced consideration. However, our discussion in the Introduction primarily references potential suppressive effects because the available literature on the impact of malaria coinfection on helminth-specific immunity is relatively limited and, to our knowledge, more frequently reports evidence consistent with suppression or modulation of helminth-associated immune responses.

3) A more detailed discussion of possible explanations (species differences, acute versus chronic infection, host age, transmission intensity, or study design) would strengthen the justification for the work

We have edited the Introduction to include these factors and their potential contribution to the inconsistent findings reported in the literature (Lines 120–126).

"The impact of malaria on helminth immune responses and clinical outcomes is likely determined by the extent to which Plasmodium-driven inflammation disrupts the Th2/regulatory milieu required for effective anti-helminth immunity. The direction and magnitude of this interaction are likely shaped by

factors such as helminth species, parasite burden, infection chronicity, host age, and transmission intensity. Inconsistencies across studies may further reflect variations in study design, diagnostic methods, and failure to control for confounding variables, such as socio-economic factors."

4) The authors should explain why malaria-induced alterations in responses to these particular antigens may be important for future vaccine development and implementation.

In the Introduction (Lines 108–115), we highlight the increasing interest in helminth vaccine development as part of elimination strategies, the challenges associated with inducing protective immunity against these complex pathogens, and the importance of a robust Th2-type immune response for an effective helminth vaccine. We further note that, given that such vaccines would likely be deployed in regions where malaria is endemic, it is important to understand whether malaria-induced immune modulation could influence responses to helminth vaccine candidate antigens and thereby potentially affect vaccine efficacy and implementation.

5) The study hypothesis is not explicitly stated: The authors should explicitly state whether they expected malaria coinfection to suppress, enhance, or otherwise modify helminth-specific immune responses

We have revised the Introduction to explicitly include a testable hypothesis (Lines 140-146).

"Given the potential impact of malaria coinfection on helminth immune responses, we hypothesised that the Th1-dominant immune environment induced by Plasmodium infection would suppress Th2-associated anti-helminth responses in children, including helminth-specific IgE and IgG4 antibody levels and type 2 cytokine concentrations, while elevating pro-inflammatory cytokines. Additionally, we hypothesised that these immunological alterations would be accompanied by worse clinical outcomes than in children with helminth infection alone."

Study design

1) The inclusion criteria remain insufficiently defined. The authors should clarify whether recruitment was consecutive, whether all eligible children were invited to participate, and how representative this population is of children living in the study area.

This study was designed as an exploratory investigation within an epidemiological project that recruited all eligible participants aged 2-10 years who presented at the Manhiça District Hospital and the Ilha Josina Machel Health Centre for clinical assessment from 2015 to 2016.

The sample may not be fully representative of the general population of children aged 2-10 years in the region, rather, it reflects children seeking care at these health facilities as explained in Lines 156-162.

2) Because the study is hospital-based rather than community-based, the study population may not be representative of the general population of helminth-infected children. Children seeking healthcare are likely to differ in disease severity, comorbidities, and socioeconomic characteristics. The authors should discuss the potential impact of selection bias on the interpretation and generalizability of the findings.

We acknowledge that the hospital-based recruitment strategy may introduce selection bias, as children attending healthcare facilities are likely to differ from those in the general community. In the Discussion section of the manuscript, we acknowledge the limitations of hospital-based recruitment and the potential for selection bias (Lines 586-590).

3) The study is cross-sectional, which limits the ability to infer temporal or causal relationships between malaria coinfection, immune responses, and anaemia. This limitation should be explicitly acknowledged and considered when interpreting the results

We acknowledge that cross-sectional design does not permit causal or temporal inference. This limitation was recognised in the Discussion section (Lines 601–602), and we have taken it into account in our interpretation of the results throughout the manuscript.

4) No sample size calculation or power analysis is provided. Given that only a small subset of participants was eventually classified as malaria–helminth coinfected, the authors should explain whether the study was adequately powered to detect differences in the numerous immunological outcomes investigated

As this study was designed as an exploratory analysis within the EcoHeMa project, a formal sample size calculation was not performed a priori, and therefore statistical power and minimum detectable effect sizes were not predefined. We acknowledge that, given the relatively small number of participants classified as malaria–helminth coinfected (n = 16), the study may have had limited statistical power to detect small or modest effects. We have explicitly stated this as a limitation in the Discussion section of the manuscript. To provide transparency regarding the precision and magnitude of estimated associations, we present regression coefficients (β) together with their corresponding 95% confidence intervals for all linear regression models. These estimates provide information on the magnitude and

direction of associations and serve as measures of effect size within the context of this exploratory analysis.

Parasite detection

1) The manuscript should clearly define how infection status was assigned. Were participants considered helminth-positive if either microscopy or qPCR was positive? Similarly, were malaria-positive individuals defined by microscopy and/or qPCR positivity? A clear diagnostic algorithm is needed to avoid ambiguity regarding participant classification

We have revised the description of the diagnostic algorithm in the manuscript (lines 305-309).

"Participant infection status was assigned using the following algorithm. For P. falciparum, children were classified as infected if they were positive by microscopy or, in the case of microscopy-negative samples, positive by qPCR. For helminths, children were classified as infected if any diagnostic method yielded a positive result. For both infections children were classified as uninfected if all diagnostic methods were negative"

2) The methods describe the detection of soil-transmitted helminths but do not specify which helminth species were ultimately included in the analyses. Given the substantial biological differences among helminths, the authors should clearly report species-specific prevalence and explain how mixed infections were handled

All helminth species detected in the study were included in the analyses, and their respective prevalences are reported in Table 1 and Figure 1B. Given the limited number of participants coinfected with malaria and helminths, we did not stratify analyses by helminth species, as this would have resulted in very small subgroup sizes and substantially reduced statistical power. Consequently, all helminth infections, including mixed-species infections, were analysed together as a single helminth-infected group. This limitation has been acknowledged in the Discussion (lines 599-601).

3) Parasite density was estimated but is not mentioned among the variables used in the analyses. Given that malaria-induced inflammation is strongly associated with parasite density, the authors should clarify whether parasite burden was evaluated as a covariate in the immunological analyses

Parasite density was estimated for P. falciparum-infected. However, it was not included as a covariate in the immunological analyses. Since parasite density is a downstream consequence of malaria infection, it can be considered as a mediator on the causal pathway between infection group and immune outcomes rather than an independent confounder. Its inclusion as a covariate would therefore

have introduced over-adjustment bias, yielding an underestimate of the total effect of malaria coinfection on helminth-specific immune responses. We note that the association between cytokine levels and P. falciparum parasitaemia has been examined in a companion analysis of the EcoHeMa cohort (Cuamba et al. 2026 BMC Medicine), and we refer the reviewer to that work for a more detailed exploration of this relationship. We acknowledge that a formal mediation analysis to look at the direct and parasitaemia-mediated effect of malaria coinfection would be of interest. However, this would require a larger coinfected sample size to be powered. We have added this point to the limitations in the Discussion section.

Statistic

The manuscript appears to involve a very large number of statistical comparisons. The authors should clearly state whether correction for multiple testing was applied (e.g., Benjamini-Hochberg FDR, Bonferroni, or other approaches). If not, the risk of false-positive associations should be discussed.

To account for multiple comparisons, we applied the Benjamini–Hochberg method. It was reported in Lines 332-333.

Results

1) The number of helminth-infected children coinfected with Plasmodium falciparum is relatively small (n = 16). Given the large number of immunological parameters evaluated (30 cytokines and multiple antibody isotypes against 11 antigens), the authors should provide a clear justification of the statistical power of the study and discuss the robustness of the findings obtained from such a limited coinfected group.

We agree that the sample size of the coinfected group is relatively small and therefore limits the statistical power of the analyses. However, we would like to reiterate that this study was designed as an exploratory analysis within a larger project, and we consider the findings to be potentially informative for the design of future, larger studies in which these observations can be further evaluated. Additionally, despite the low sample size, we detect statistically significant associations. We have acknowledged these limitations in the Discussion and have interpreted the results with appropriate caution throughout the manuscript.

2) The term “helminth infection” encompasses parasites with markedly different immunological profiles. The authors should specify the distribution of helminth species and assess whether the

observed associations are driven by particular species or remain consistent after species-specific adjustment.

We agree that helminth species differ in their immunological profiles and that species-specific effects may influence observed associations. However, due to the relatively small number of coinfected participants, species-specific stratified analyses or species-adjusted models were not feasible, as this would have resulted in very small subgroup sizes and limited statistical power. We have clarified this limitation in the Discussion and acknowledge that future studies with larger sample sizes should ideally address species-specific effects more explicitly.

3) The authors report associations between cytokine concentrations and demographic/clinical variables based on univariable analyses only. Given the potential interrelationships among age, infection status, anaemia, body temperature, and other clinical variables, it would be informative to present multivariable analyses to assess whether these associations remain independent after adjustment for potential confounders.

In the present study, analyses examining associations between cytokine concentrations and demographic/clinical variables were performed using univariable approaches, as they were exploratory and aimed to describe potential patterns of association.

In contrast, the main analyses assessing the impact of coinfection on cytokine responses were conducted using multivariable linear regression models, as well, in which we adjusted for potential confounders (including age, sex, and area of residence) based on prior knowledge and directed acyclic graph (DAG) informed confounder assessment (Line 330-332).

4) The magnitude of several reported associations is remarkably large, with percentage increases exceeding 100% for some antibody responses and reaching 172% for total IgE. Given the limited number of coinfected participants (n = 16), the authors should demonstrate that these findings are not driven by a small number of influential observations.

During the analysis and interpretation of the results, we examined individual scatterplots to assess whether the observed associations were disproportionately influenced by outliers or a small number of observations. Representative scatterplots for the most relevant findings are presented in the manuscript to allow readers to evaluate the distribution of the data. Given the large number of immunological parameters analysed, it was not feasible to include scatterplots and boxplots for all associations.

Nevertheless, we acknowledge that the relatively small number of coinfected participants may have influenced the statistical power and stability of some estimates. To aid interpretation, all effect estimates are presented together with their 95% confidence intervals, which reflect the uncertainty associated with the sample size. This limitation has been acknowledged in the Discussion, and the findings have been interpreted with appropriate caution.

Discussion

1) The Discussion focuses primarily on malaria coinfection as the driver of the observed immune differences. However, alternative explanations, including variation in malaria parasite density, helminth burden, or polyparasitism, receive limited attention. These factors should be discussed more thoroughly as potential contributors to the observed associations

We did not discuss these factors in detail because helminth burden was not measured and P. falciparum parasite density was not included in the analyses. In addition, the limited sample size precluded meaningful analyses of polyparasitism. However, we agree that these factors may influence immune responses and could therefore have contributed to the observed associations. We have acknowledged this as a limitation in the Discussion and note that we cannot exclude the possibility that variation in parasite burden or polyparasitism influenced our findings.

2) The statement that coinfected and helminth-only children exhibit distinct immune profiles should be interpreted cautiously. Given the small size of the coinfected group, the risk of overfitting should be explicitly acknowledged.

We have revised the text to provide a more cautious interpretation (Lines 576-577).

3) The potential implications for helminth vaccine efficacy are interesting but remain hypothetical. The Discussion should avoid overextending the findings beyond the available data and clearly state that vaccine-related implications are speculative.

We have revised the wording regarding the potential implications of malaria coinfection for helminth vaccine efficacy to avoid overly affirmative statements. We have also acknowledged the limitation of the small sample size in the Discussion.

Reviewer #3: I am very happy with the responses of the authors and their changes made in response to reviewer comments.

I congratulate the authors on a great piece of science!

We thank the reviewer for the positive assessment of our revised manuscript and for the helpful feedback provided during the review process.
