# Supplementary material for: Helminth-infected Mozambican children with malaria have increased anaemia, cytokines and helminth-specific antibodies
Source: PLoS Negl Trop Dis. 2026 Jul 13;20(7):e0014485. doi: 10.1371/journal.pntd.0014485 (PMC13362103; doi:10.1371/journal.pntd.0014485)
Supplement: Peer review history

# Peer review: Decision Letter 0

**DOI:** 10.1371/journal.pntd.0014485.r001

**Authors:** Krystyna Cwiklinski, Chukwunonso Nzelu

25 Mar 2026

PNTD-D-26-00029

Helminth-infected Mozambican children with malaria have increased anaemia, cytokines and helminth-specific antibodies

PLOS Neglected Tropical Diseases

Dear Cuamba,

Thank you for submitting your manuscript to PLOS Neglected Tropical Diseases. After careful consideration, we feel that it has merit but does not fully meet PLOS Neglected Tropical Diseases's publication criteria as it currently stands. Therefore, we invite you to submit a revised version of the manuscript that addresses the points raised during the review process.

Please submit your revised manuscript by May 24 2026 11:59PM. If you will need more time than this to complete your revisions, please reply to this message or contact the journal office at plosntds@plos.org. When you're ready to submit your revision, log on to https://www.editorialmanager.com/pntd/ and select the 'Submissions Needing Revision' folder to locate your manuscript file.

Please include the following items when submitting your revised manuscript:

* A letter that responds to each point raised by the editor and reviewer(s). You should upload this letter as a separate file labeled 'Response to Reviewers'. This file does not need to include responses to any formatting updates and technical items listed in the 'Journal Requirements' section below.

* A marked-up copy of your manuscript that highlights changes made to the original version. You should upload this as a separate file labeled 'Revised Manuscript with Track Changes'.

* An unmarked version of your revised paper without tracked changes. You should upload this as a separate file labeled 'Manuscript'.

If you would like to make changes to your financial disclosure, competing interests statement, or data availability statement, please make these updates within the submission form at the time of resubmission. Guidelines for resubmitting your figure files are available below the reviewer comments at the end of this letter.

We look forward to receiving your revised manuscript.

Kind regards,

Chukwunonso Nzelu, Ph.D.

Academic Editor

PLOS Neglected Tropical Diseases

-->Krystyna Cwiklinski

Section Editor

PLOS Neglected Tropical Diseases

Shaden Kamhawi

co-Editor-in-Chief

PLOS Neglected Tropical Diseases

orcid.org/0000-0003-4304-636XX

Paul Brindley

co-Editor-in-Chief

PLOS Neglected Tropical Diseases

orcid.org/0000-0003-1765-0002

**Journal Requirements:**

1) Please ensure that the CRediT author contributions listed for every co-author are completed accurately and in full.

At this stage, the following Authors/Authors require contributions: Inocencia Cuamba, Rebeca Santano, Berta Grau-Pujol, Marta Vidal, Anélsio Cossa, Chenjerai Jairoce, Rojelio Mejía, José Muñoz, Ruth Aguilar, Bin Zhan, Augusto Nhabomba, Gemma Moncunill, and Carlota Dobaño. Please ensure that the full contributions of each author are acknowledged in the "Add/Edit/Remove Authors" section of our submission form.

The list of CRediT author contributions may be found here: https://journals.plos.org/plosntds/s/authorship#loc-author-contributions

2) We do not publish any copyright or trademark symbols that usually accompany proprietary names, eg ©,  ®, or TM  (e.g. next to drug or reagent names). Therefore please remove all instances of trademark/copyright symbols throughout the text, including:

- ® on page: 9 and 25.

3) In the online submission form, you indicated that "The data supporting the findings of this study are available from the corresponding author upon reasonable request.". All PLOS journals now require all data underlying the findings described in their manuscript to be freely available to other researchers, either

1. In a public repository

2. Within the manuscript itself

3. Uploaded as supplementary information.

This policy applies to all data except where public deposition would breach compliance with the protocol approved by your research ethics board. If your data cannot be made publicly available for ethical or legal reasons (e.g., public availability would compromise patient privacy), please explain your reasons by return email and your exemption request will be escalated to the editor for approval. Your exemption request will be handled independently and will not hold up the peer review process, but will need to be resolved should your manuscript be accepted for publication. One of the Editorial team will then be in touch if there are any issues.

**Reviewers' comments:**

Reviewer's Responses to Questions

**Key Review Criteria Required for Acceptance?**

As you describe the new analyses required for acceptance, please consider the following:

**Methods**

-Are the objectives of the study clearly articulated with a clear testable hypothesis stated?

-Is the study design appropriate to address the stated objectives?

-Is the population clearly described and appropriate for the hypothesis being tested?

-Is the sample size sufficient to ensure adequate power to address the hypothesis being tested?

-Were correct statistical analysis used to support conclusions?

-Are there concerns about ethical or regulatory requirements being met?

Reviewer #1: no

yes

no

yes

yes

no

Reviewer #2: The study objectives are clearly articulated, with a well-defined and testable hypothesis regarding the impact of malaria coinfection on helminth-specific immunity and clinical outcomes. The cross-sectional, hospital-based design is generally appropriate to explore the stated research questions, although, as acknowledged by the authors, it does not allow for causal inferences. The study population is well described, including age range, recruitment sites, inclusion and exclusion criteria, and relevant clinical characteristics, and is appropriate for addressing the objectives. The overall sample size is reasonable; however, the small number of coinfected participants (n=16) may limit statistical power and increase variability in effect estimates. The statistical analyses, including multivariable regression models and interaction terms, are appropriate for examining cytokine–antibody relationships, with consideration given to multiple comparisons and the exploratory nature of some analyses. Ethical and regulatory requirements were met, with approvals obtained from both the Mozambican National Bioethics Committee and the Spanish Clinical Research Ethics Committee, and written informed consent was obtained from all parents or guardians.

Reviewer #3: The objectives of the study are clearly stated. The design and population is appropriate for the hypothesis tested.

The sample size is small (with respect to the 16 co-infected participants), but this is addressed as a limitation in the discussion and clearly stated throughout. The statistical analysis seems robust. Ethical requirements seem appropriate.

A couple of small things:

- In Table 1 there are some missing participants (e.g. only 86-89 participants) for some of the clinical outcomes of fever etc. Please can you provide information in the methods as to why these participants are missing these outcomes, and whether there could be any bias in this data loss. Also – in the figures you say N=96, this is inaccurate in those (e.g. fig 2) when clinical outcomes are used for which you only have N=87.

- Line 186 the chemokine RANTES was excluded due to a substantial number of samples above the ULOQ. Can you quantify “substantial”? Are there any cytokines with a substantial number below the LLOQ, can you provide information on this here?

- Can you fit 200ul in a 374 well plate (line 215)?

- Were samples randomised between plates?

**********

**Results**

-Does the analysis presented match the analysis plan?

-Are the results clearly and completely presented?

-Are the figures (Tables, Images) of sufficient quality for clarity?

Reviewer #1: yes

yes

no

Reviewer #2: The analyses presented are consistent with the methods described in the manuscript and align with the stated study objectives, including the use of multivariable regression, interaction terms, hierarchical clustering, and multivariate approaches. The results are clearly and comprehensively presented, with detailed comparisons between helminth-only and coinfected children that allow readers to follow the key findings. Figures and tables are high quality, clearly labeled, and effectively illustrate the study findings.

Reviewer #3: The results are clearly and completely presented. The figures are high-quality and clear.

I am missing any view of the underlying data for the models. Would it be possible to include a supplementary figure(s) with more traditional plots (e.g. boxplot/volcano with individual points per participant) showing helminth vs coinfected? If all outputs is too much, perhaps just including key (significant?) findings.

- When you state the p value in the results are you always referring to the adjusted p? Could you state this explicitly in the text?

- In Fig 5 there is a misaligned extra purple square on top of IL8/IgG1

**********

**Conclusions**

-Are the conclusions supported by the data presented?

-Are the limitations of analysis clearly described?

-Do the authors discuss how these data can be helpful to advance our understanding of the topic under study?

-Is public health relevance addressed?

Reviewer #1: yes

no

yes

yes

Reviewer #2: The conclusions drawn in the manuscript are well supported by the data presented, reflecting the observed associations between malaria coinfection, altered cytokine and antibody responses, and clinical outcomes such as anemia. The limitations of the analysis, including the small number of coinfected participants, the cross-sectional design, and the inability to stratify by helminth species, are clearly acknowledged

Reviewer #3: The conclusions are supported by the data presented, and the discussion is good-quality. Limitations and advances of the study are discussed.

**********

**Editorial and Data Presentation Modifications?**

Use this section for editorial suggestions as well as relatively minor modifications of existing data that would enhance clarity. If the only modifications needed are minor and/or editorial, you may wish to recommend “Minor Revision” or “Accept”.

Reviewer #1: The proposal is well designed scientifically and methodologically. But I have jot down some comments and suggestions

On the title:

There are several studies conducted on similar topic, what is novelty and the values that add the previous study?

In the case of co-infection with malaria and STH infection

How do you differentiate whether malaria or STH cause anemia

How do you measure the severity of the anemia beyond Hgb level or hematocrit level?

I am not comfortable with the naming of the title, it seems like a conclusion to me.

Method and materials

Describe the catchment area of each health facility?

Please describe the current situation of malaria and STHs in the study area.

You have not clearly stated your sample size calculation and determination?

How did you allocate your sample size to the two health facilities?

Why you did only included children between the age of 2 and 10 for the study?

How did you control cofounding variables b/c Hgb is subjected to confounding variables?

Give details of statistical considerations in sample size considerations. Also have you checked the effect size?

Have you calculated additional percentage for your sample size to take care of drop out?

Why you did not include healthy controls?

Why didn’t you perform STHs parasite intensity analysis with the severity of anemia?

Why didn’t you make the study as comparative cross-sectional study?

Reviewer #2: (No Response)

Reviewer #3: (No Response)

**********

**Summary and General Comments**

Use this section to provide overall comments, discuss strengths/weaknesses of the study, novelty, significance, general execution and scholarship. You may also include additional comments for the author, including concerns about dual publication, research ethics, or publication ethics. If requesting major revision, please articulate the new experiments that are needed.

Reviewer #1: None

Reviewer #2: (No Response)

Reviewer #3: This is a technically impressive and well-executed study, with high-quality visualisations and analyses. Whilst the low sample size (n=16 for coinfected) is a clear limitation, it is understandable that large sample sizes may be difficult with the depth of immune outputs analysed. Whilst this study extends and deepens our understanding of responses in co-infected individuals, it does not represent a large ‘leap’ forward in conceptual understanding. This is discussed further below.

A key area where I think this study is lacking is in not including a malaria-only group. I believe it is well known that acute malaria leads to anaemia and alterations in circulating cytokines. Is it relevant that these individuals have helminth infection, or would these outcomes be exactly the same if comparing acute malaria to no malaria in a helminth uninfected population? Were they outputs (e.g. cytokines, clinical symptoms such as anaemia) run on this group? If so it would greatly improve the novelty of the study to include.

Note – the above criticism is less applicable to helminth-specific antibody outputs (although still interesting – presumably these children could have helminth-specific antibodies from a prior helminth infection, does malaria increase those? This may fit your polyclonal b cell activation hypothesis (line 517).)

The authors have previously shown that malaria co-infection increases helminth-specific antibodies in coinfected individuals (ref 50). I am surprised this reference appears so late in the text, and should be highlighted in the introduction. The authors should be more explicit as to how the current work extends the findings of ref 50 (and other relevant literature?)– e.g. isotypes? Study population? antigens tested?

**********

PLOS authors have the option to publish the peer review history of their article (what does this mean?). If published, this will include your full peer review and any attached files.

If you choose “no”, your identity will remain anonymous but your review may still be made public.

**Do you want your identity to be public for this peer review?**  For information about this choice, including consent withdrawal, please see our Privacy Policy.

Reviewer #1: No

Reviewer #2: No

Reviewer #3: No

[NOTE: If reviewer comments were submitted as an attachment file, they will be attached to this email and accessible via the submission site. Please log into your account, locate the manuscript record, and check for the action link "View Attachments". If this link does not appear, there are no attachment files.]

**Figure resubmission:**

While revising your submission, we strongly recommend that you use PLOS’s NAAS tool (https://ngplosjournals.pagemajik.ai/artanalysis) to test your figure files. NAAS can convert your figure files to the TIFF file type and meet basic requirements (such as print size, resolution), or provide you with a report on issues that do not meet our requirements and that NAAS cannot fix.

After uploading your figures to PLOS’s NAAS tool - https://ngplosjournals.pagemajik.ai/artanalysis, NAAS will process the files provided and display the results in the "Uploaded Files" section of the page as the processing is complete. If the uploaded figures meet our requirements (or NAAS is able to fix the files to meet our requirements), the figure will be marked as "fixed" above. If NAAS is unable to fix the files, a red "failed" label will appear above. When NAAS has confirmed that the figure files meet our requirements, please download the file via the download option, and include these NAAS processed figure files when submitting your revised manuscript.

**Reproducibility:**

To enhance the reproducibility of your results, we recommend that authors of applicable studies deposit laboratory protocols in protocols.io, where a protocol can be assigned its own identifier (DOI) such that it can be cited independently in the future. Additionally, PLOS ONE offers an option to publish peer-reviewed clinical study protocols. Read more information on sharing protocols at https://plos.org/protocols?utm_medium=editorial-email&utm_source=authorletters&utm_campaign=protocols

Attachment Submitted filename: Review_Comments.pdf

# Author comment: Author response to Decision Letter 1

**DOI:** 10.1371/journal.pntd.0014485.r002

4 May 2026

Attachment Submitted filename: Response to Reviewers.pdf

# Peer review: Decision Letter 1

**DOI:** 10.1371/journal.pntd.0014485.r003

**Authors:** Krystyna Cwiklinski, Chukwunonso Nzelu, Krystyna Cwiklinski, Chukwunonso Nzelu

1 Jun 2026

PNTD-D-26-00029R1

Helminth-infected Mozambican children with malaria have increased anaemia, cytokines and helminth-specific antibodies

PLOS Neglected Tropical Diseases

Dear Dr. Dobano and Cuamba,

Thank you for submitting your manuscript to PLOS Neglected Tropical Diseases. After careful consideration, we feel that it has merit but does not fully meet PLOS Neglected Tropical Diseases's publication criteria as it currently stands. Therefore, we invite you to submit a revised version of the manuscript that addresses the points raised during the review process.

Please submit your revised manuscript by Jul 31 2026 11:59PM. If you will need more time than this to complete your revisions, please reply to this message or contact the journal office at plosntds@plos.org. When you're ready to submit your revision, log on to https://www.editorialmanager.com/pntd/ and select the 'Submissions Needing Revision' folder to locate your manuscript file.

Please include the following items when submitting your revised manuscript:

* A letter that responds to each point raised by the editor and reviewer(s). You should upload this letter as a separate file labeled 'Response to Reviewers'. This file does not need to include responses to any formatting updates and technical items listed in the 'Journal Requirements' section below.

-->* A marked-up copy of your manuscript that highlights changes made to the original version. You should upload this as a separate file labeled 'Revised Manuscript with Track Changes'.

* An unmarked version of your revised paper without tracked changes. You should upload this as a separate file labeled 'Manuscript'.

-->If you would like to make changes to your financial disclosure, competing interests statement, or data availability statement, please make these updates within the submission form at the time of resubmission. Guidelines for resubmitting your figure files are available below the reviewer comments at the end of this letter.

As the corresponding author, your ORCID iD is verified in the submission system and will appear in the published article. PLOS supports the use of ORCID, and we encourage all coauthors to register for an ORCID iD and use it as well. Please encourage your coauthors to verify their ORCID iD within the submission system before final acceptance, as unverified ORCID iDs will not appear in the published article. Only the individual author can complete the verification step; PLOS staff cannot verify ORCID iDs on behalf of authors.

We look forward to receiving your revised manuscript.

Kind regards,

Chukwunonso Nzelu, Ph.D.

Academic Editor

PLOS Neglected Tropical Diseases

Krystyna Cwiklinski

Section Editor

PLOS Neglected Tropical Diseases

Shaden Kamhawi

co-Editor-in-Chief

PLOS Neglected Tropical Diseases

orcid.org/0000-0003-4304-636XX

Paul Brindley

co-Editor-in-Chief

PLOS Neglected Tropical Diseases

orcid.org/0000-0003-1765-0002

**Journal Requirements:**

If the reviewer comments include a recommendation to cite specific previously published works, please review and evaluate these publications to determine whether they are relevant and should be cited. There is no requirement to cite these works unless the editor has indicated otherwise.

**Reviewers' comments:**

Reviewer's Responses to Questions

**Key Review Criteria Required for Acceptance?**

As you describe the new analyses required for acceptance, please consider the following:

**Methods**

-Are the objectives of the study clearly articulated with a clear testable hypothesis stated?

-Is the study design appropriate to address the stated objectives?

-Is the population clearly described and appropriate for the hypothesis being tested?

-Is the sample size sufficient to ensure adequate power to address the hypothesis being tested?

-Were correct statistical analysis used to support conclusions?

-Are there concerns about ethical or regulatory requirements being met?

Reviewer #2: The study addresses an important question on malaria–helminth coinfection and immune responses in children. The objectives are relevant, but a clear testable hypothesis is lacking. The cross-sectional design is appropriate for associations but does not allow causal inference. The hospital-based recruitment may introduce selection bias. A major limitation is the very small number of coinfected participants (n = 16), raising concerns about statistical power and robustness of the analyses. Although appropriate statistical methods were used, further clarification is needed regarding adjustment for key confounders. Overall, the study is of interest but methodological and statistical limitations should be addressed before firm conclusions can be drawn.

Reviewer #3: (No Response)

**********

**Results**

-Does the analysis presented match the analysis plan?

-Are the results clearly and completely presented?

-Are the figures (Tables, Images) of sufficient quality for clarity?

Reviewer #2: The analyses generally appear consistent with the statistical methods described. The results are clearly presented overall, although a clearer distinction between findings that remain significant after multiple-testing correction and exploratory findings would improve interpretation. A major concern remains the small number of coinfected participants, which may affect the robustness of some analyses. In addition, the quality of several figures is suboptimal. The image resolution should be improved, as some text, labels, and graphical elements are difficult to read. Higher-resolution figures are needed to ensure clarity and proper evaluation of the results.

Reviewer #3: (No Response)

**********

**Conclusions**

-Are the conclusions supported by the data presented?

-Are the limitations of analysis clearly described?

-Do the authors discuss how these data can be helpful to advance our understanding of the topic under study?

-Is public health relevance addressed?

Reviewer #2: The conclusions are generally supported by the data but are somewhat stronger than warranted given the cross-sectional design and the small number of coinfected participants. The limitations are acknowledged, but key issues such as potential confounding and limited statistical power could be more fully emphasized. The authors discuss the relevance of the findings for understanding malaria–helminth interactions, and the public health implications are mentioned, although they remain largely speculative and should be toned down.

Reviewer #3: (No Response)

**********

**Editorial and Data Presentation Modifications?**

Use this section for editorial suggestions as well as relatively minor modifications of existing data that would enhance clarity. If the only modifications needed are minor and/or editorial, you may wish to recommend “Minor Revision” or “Accept”.

Reviewer #2: (No Response)

Reviewer #3: (No Response)

**********

**Summary and General Comments**

Use this section to provide overall comments, discuss strengths/weaknesses of the study, novelty, significance, general execution and scholarship. You may also include additional comments for the author, including concerns about dual publication, research ethics, or publication ethics. If requesting major revision, please articulate the new experiments that are needed.

Reviewer #2: The topic is relevant and original. The study addresses a relatively underexplored question: the impact of malaria co-infection on anti-helminth immune responses in children. The immunological approach is ambitious, and the results appear to be interesting. However, several points require clarification.

Introduction

1) The authors should moderate this statement and more clearly define the specific gap addressed by the present study (e.g., effects on antibody responses against vaccine candidate antigens and multiplex immune profiling in children)

2) The introduction would benefit from a more balanced discussion of the potential mechanisms underlying both suppressive and enhancing effects of malaria coinfection

3) A more detailed discussion of possible explanations (species differences, acute versus chronic infection, host age, transmission intensity, or study design) would strengthen the justification for the work

4) The authors should explain why malaria-induced alterations in responses to these particular antigens may be important for future vaccine development and implementation

5) The study hypothesis is not explicitly stated: The authors should explicitly state whether they expected malaria coinfection to suppress, enhance, or otherwise modify helminth-specific immune responses

Study design

1) The inclusion criteria remain insufficiently defined. The authors should clarify whether recruitment was consecutive, whether all eligible children were invited to participate, and how representative this population is of children living in the study area

2) Because the study is hospital-based rather than community-based, the study population may not be representative of the general population of helminth-infected children. Children seeking healthcare are likely to differ in disease severity, comorbidities, and socioeconomic characteristics. The authors should discuss the potential impact of selection bias on the interpretation and generalizability of the findings.

3) The study is cross-sectional, which limits the ability to infer temporal or causal relationships between malaria coinfection, immune responses, and anaemia. This limitation should be explicitly acknowledged and considered when interpreting the results

4) No sample size calculation or power analysis is provided. Given that only a small subset of participants was eventually classified as malaria–helminth coinfected, the authors should explain whether the study was adequately powered to detect differences in the numerous immunological outcomes investigated

Parasite detection

1) The manuscript should clearly define how infection status was assigned. Were participants considered helminth-positive if either microscopy or qPCR was positive? Similarly, were malaria-positive individuals defined by microscopy and/or qPCR positivity? A clear diagnostic algorithm is needed to avoid ambiguity regarding participant classification

2) The methods describe the detection of soil-transmitted helminths but do not specify which helminth species were ultimately included in the analyses. Given the substantial biological differences among helminths, the authors should clearly report species-specific prevalence and explain how mixed infections were handled

3) Parasite density was estimated but is not mentioned among the variables used in the analyses. Given that malaria-induced inflammation is strongly associated with parasite density, the authors should clarify whether parasite burden was evaluated as a covariate in the immunological analyses

Statistic

The manuscript appears to involve a very large number of statistical comparisons. The authors should clearly state whether correction for multiple testing was applied (e.g., Benjamini-Hochberg FDR, Bonferroni, or other approaches). If not, the risk of false-positive associations should be discussed.

Results

1) The number of helminth-infected children coinfected with Plasmodium falciparum is relatively small (n = 16). Given the large number of immunological parameters evaluated (30 cytokines and multiple antibody isotypes against 11 antigens), the authors should provide a clear justification of the statistical power of the study and discuss the robustness of the findings obtained from such a limited coinfected group.

2) The term “helminth infection” encompasses parasites with markedly different immunological profiles. The authors should specify the distribution of helminth species and assess whether the observed associations are driven by particular species or remain consistent after species-specific adjustment.

3) The authors report associations between cytokine concentrations and demographic/clinical variables based on univariable analyses only. Given the potential interrelationships among age, infection status, anaemia, body temperature, and other clinical variables, it would be informative to present multivariable analyses to assess whether these associations remain independent after adjustment for potential confounders

4) The magnitude of several reported associations is remarkably large, with percentage increases exceeding 100% for some antibody responses and reaching 172% for total IgE. Given the limited number of coinfected participants (n = 16), the authors should demonstrate that these findings are not driven by a small number of influential observations

Discussion

1) The Discussion focuses primarily on malaria coinfection as the driver of the observed immune differences. However, alternative explanations, including variation in malaria parasite density, helminth burden, or polyparasitism, receive limited attention. These factors should be discussed more thoroughly as potential contributors to the observed associations

2) The statement that coinfected and helminth-only children exhibit distinct immune profiles should be interpreted cautiously. Given the small size of the coinfected group, the risk of overfitting should be explicitly acknowledged

3) The potential implications for helminth vaccine efficacy are interesting but remain hypothetical. The Discussion should avoid overextending the findings beyond the available data and clearly state that vaccine-related implications are speculative.

Reviewer #3: I am very happy with the responses of the authors and their changes made in response to reviewer comments.

I congratulate the authors on a great piece of science!

**********

PLOS authors have the option to publish the peer review history of their article (what does this mean?). If published, this will include your full peer review and any attached files.

If you choose “no”, your identity will remain anonymous but your review may still be made public.

**Do you want your identity to be public for this peer review?**  For information about this choice, including consent withdrawal, please see our Privacy Policy.

Reviewer #2: No

Reviewer #3: No

[NOTE: If reviewer comments were submitted as an attachment file, they will be attached to this email and accessible via the submission site. Please log into your account, locate the manuscript record, and check for the action link "View Attachments". If this link does not appear, there are no attachment files.]

**Figure resubmission:**

While revising your submission, we strongly recommend that you use PLOS’s NAAS tool (https://ngplosjournals.pagemajik.ai/artanalysis) to test your figure files. NAAS can convert your figure files to the TIFF file type and meet basic requirements (such as print size, resolution), or provide you with a report on issues that do not meet our requirements and that NAAS cannot fix.

After uploading your figures to PLOS’s NAAS tool - https://ngplosjournals.pagemajik.ai/artanalysis, NAAS will process the files provided and display the results in the "Uploaded Files" section of the page as the processing is complete. If the uploaded figures meet our requirements (or NAAS is able to fix the files to meet our requirements), the figure will be marked as "fixed" above. If NAAS is unable to fix the files, a red "failed" label will appear above. When NAAS has confirmed that the figure files meet our requirements, please download the file via the download option, and include these NAAS processed figure files when submitting your revised manuscript.

**Reproducibility:**

To enhance the reproducibility of your results, we recommend that authors of applicable studies deposit laboratory protocols in protocols.io, where a protocol can be assigned its own identifier (DOI) such that it can be cited independently in the future. Additionally, PLOS ONE offers an option to publish peer-reviewed clinical study protocols. Read more information on sharing protocols at https://plos.org/protocols?utm_medium=editorial-email&utm_source=authorletters&utm_campaign=protocols

# Author comment: Author response to Decision Letter 2

**DOI:** 10.1371/journal.pntd.0014485.r004

11 Jun 2026

Attachment Submitted filename: Response_to_Reviewers_auresp_2.pdf

# Editor report: Decision Letter 2

**DOI:** 10.1371/journal.pntd.0014485.r005

**Authors:** Krystyna Cwiklinski, Chukwunonso Nzelu, Krystyna Cwiklinski, Chukwunonso Nzelu, Krystyna Cwiklinski, Chukwunonso Nzelu

19 Jun 2026

Dear Cuamba,

We are pleased to inform you that your manuscript 'Helminth-infected Mozambican children with malaria have increased anaemia, cytokines and helminth-specific antibodies' has been provisionally accepted for publication in PLOS Neglected Tropical Diseases.

Before your manuscript can be formally accepted you will need to complete some formatting changes, which you will receive in a follow up email. A member of our team will be in touch with a set of requests.

Please note that your manuscript will not be scheduled for publication until you have made the required changes, so a swift response is appreciated.

IMPORTANT: The editorial review process is now complete. PLOS will only permit corrections to spelling, formatting or significant scientific errors from this point onwards. Requests for major changes, or any which affect the scientific understanding of your work, will cause delays to the publication date of your manuscript.

Should you, your institution's press office or the journal office choose to press release your paper, you will automatically be opted out of early publication. We ask that you notify us now if you or your institution is planning to press release the article. All press must be co-ordinated with PLOS.

Thank you again for supporting Open Access publishing; we are looking forward to publishing your work in PLOS Neglected Tropical Diseases.

Best regards,

Chukwunonso Nzelu, Ph.D.

Academic Editor

PLOS Neglected Tropical Diseases

Krystyna Cwiklinski

Section Editor

PLOS Neglected Tropical Diseases

Shaden Kamhawi

co-Editor-in-Chief

PLOS Neglected Tropical Diseases

orcid.org/0000-0003-4304-636XX

Paul Brindley

co-Editor-in-Chief

PLOS Neglected Tropical Diseases

orcid.org/0000-0003-1765-0002

***********************************************************

p.p1 {margin: 0.0px 0.0px 0.0px 0.0px; line-height: 16.0px; font: 14.0px Arial; color: #323333; -webkit-text-stroke: #323333}span.s1 {font-kerning: none

# Editor report: Acceptance letter

**DOI:** 10.1371/journal.pntd.0014485.r006

**Authors:** Krystyna Cwiklinski, Chukwunonso Nzelu, Krystyna Cwiklinski, Chukwunonso Nzelu, Krystyna Cwiklinski, Chukwunonso Nzelu

Dear Cuamba,

We are delighted to inform you that your manuscript, "

Helminth-infected Mozambican children with malaria have increased anaemia, cytokines and helminth-specific antibodies," has been formally accepted for publication in PLOS Neglected Tropical Diseases.

We have now passed your article onto the PLOS Production Department who will complete the rest of the publication process. All authors will receive a confirmation email upon publication.

The corresponding author will soon be receiving a typeset proof for review, to ensure errors have not been introduced during production. Please review the PDF proof of your manuscript carefully, as this is the last chance to correct any scientific or type-setting errors. Please note that major changes, or those which affect the scientific understanding of the work, will likely cause delays to the publication date of your manuscript. Note: Proofs for Front Matter articles (Editorial, Viewpoint, Symposium, Review, etc...) are generated on a different schedule and may not be made available as quickly.

Soon after your final files are uploaded, the early version of your manuscript will be published online unless you opted out of this process. The date of the early version will be your article's publication date. The final article will be published to the same URL, and all versions of the paper will be accessible to readers.

For Research Articles, you will receive an invoice from PLOS for your publication fee after your manuscript has reached the completed accept phase. If you receive an email requesting payment before acceptance or for any other service, this may be a phishing scheme. Learn how to identify phishing emails and protect your accounts at https://explore.plos.org/phishing.

Thank you again for supporting open-access publishing; we are looking forward to publishing your work in PLOS Neglected Tropical Diseases.

Best regards,

Shaden Kamhawi

co-Editor-in-Chief

PLOS Neglected Tropical Diseases

Paul Brindley

co-Editor-in-Chief

PLOS Neglected Tropical Diseases
